# Formation, Characterization, and Bonding of *cis*- and *trans*-[PtCl_2_{Te(CH_2_)_6_}_2_], *cis-trans*-[Pt_3_Cl_6_{Te(CH_2_)_6_}_4_], and *cis*-*trans*-[Pt_4_Cl_8_{Te(CH_2_)_6_}_4_]: Experimental and DFT Study †

**DOI:** 10.3390/molecules28227551

**Published:** 2023-11-12

**Authors:** Marko Rodewald, J. Mikko Rautiainen, Helmar Görls, Raija Oilunkaniemi, Wolfgang Weigand, Risto S. Laitinen

**Affiliations:** 1 Institute for Inorganic and Analytical Chemistry, Friedrich Schiller University of Jena, Humboldt Str. 8, 07743 Jena, Germany; marko.rodewald@uni-jena.de (M.R.); helmar.goerls@uni-jena.de (H.G.); 2 Department of Chemistry and Nanoscience Center, University of Jyväskylä, P.O. Box 35, 40014 Jyväskylä, Finland; j.mikko.rautiainen@jyu.fi; 3 Laboratory of Inorganic Chemistry, Environmental and Chemical Engineering, University of Oulu, P.O. Box 3000, 90014 Oulu, Finland; raija.oilunkaniemi@oulu.fi

**Keywords:** density functional calculations, NMR spectroscopy, platinum, tellurium, X-ray diffraction

## Abstract

[PtCl_2_{Te(CH_2_)_6_}_2_] (**1**) was synthesized from the cyclic telluroether Te(CH_2_)_6_ and *cis*-[PtCl_2_(NCPh)_2_] in dichloromethane at room temperature under the exclusion of light. The crystal structure determination showed that in the solid state, **1** crystallizes as yellow plate-like crystals of the *cis*-isomer **1*_cis_*** and the orange-red interwoven needles of **1*_trans_***. The crystals could be separated under the microscope. NMR experiments showed that upon dissolution of the crystals of **1*_cis_*** in CDCl_3_, it isomerizes and forms a dynamic equilibrium with the *trans*-isomer **1*_trans_*** that becomes the predominant species. Small amounts of *cis-trans*-[Pt_3_Cl_6_{Te(CH_2_)_6_}_4_] (**2**) and *cis*-*trans*-[Pt_4_Cl_8_{Te(CH_2_)_6_}_4_] (**3**) were also formed and structurally characterized. Both compounds show rare bridging telluroether ligands and two different platinum coordination environments, one exhibiting a *cis*-Cl/*cis*-Te(CH_2_)_6_ arrangement and the other a *trans*-Cl/*trans*-Te(CH_2_)_6_ arrangement. Complex **2** has an open structure with two terminal and two bridging telluroether ligands, whereas complex **3** has a cyclic structure with four Te(CH_2_)_6_ bridging ligands. The bonding and formation of the complexes have been discussed through the use of DFT calculations combined with QTAIM analysis. The recrystallization of the mixture of the 1:1 reaction from *d*_6_-DMSO afforded [PtCl_2_{S(O)(CD_3_)_2_}{Te(CH_2_)_6_}] (**4**) that could also be characterized both structurally and spectroscopically.

## 1. Introduction 

The advent of the versatile chemistry of crown-ethers and their complexes in the 1980s [1,2] has generated interest in the related macrocycles of heavier chalcogen elements (for some recent reviews, see refs. [3,4,5,6,7,8,9,10,11,12,13,14,15,16,17,18]). The information on the syntheses, structures, and coordination chemistry of thioethers is particularly extensive, but the related seleno- and telluroethers have also shown growing research activity in recent decades. The study of macrocyclic chalcogen heterocycles helps to gain insight into the chalcogen bonding and its applications in supramolecular chemistry and crystal engineering [19,20,21,22,23,24,25]. The chalcogen bonding interactions are most significant in the case of tellurium. 

The information on cyclic saturated telluroethers is sparse compared to related thioethers and selenoethers. While the preparation of cyclic Te(CH_2_)_4_ [26], Te(CH_2_)_5_ [27], and 1,5,9-Te_3_(CH_2_)_9_ [28] has been known for a long time, it is only recently that the crystal structures and bonding in some [Te(CH_2_)*_m_*]*_n_* (*n* = 1–4, *m* = 3–7) [29] have been discussed. The coordination chemistry of the cyclic telluroethers is also little studied with only [MCl_2_{Te(CH_2_)_4_}_2_] (M = Pd, Pt) [30], [PtCl_2_{Te(CH_2_)_4_O}_2_] [31], [MCl_2_{Te_2_O_4_(CH_2_)_12_}] (M = Pd. Pt) [32], [Rh_2_(*η*^5^-C_5_H_5_)_2_(CO)(*μ-η^1^:η^1^*- CF_3_C_2_CF_3_){Te(CH_2_)_4_}] [33], and [Ag{TeS_2_(CH_2_)_8_}]*_n_*[BF_4_]*_n_* [34] being either structurally or spectroscopically characterized.

The current contribution is the continuation of our systematic investigation of the synthesis of the series of telluroether heterocycles [Te(CH_2_)*_m_*]*_n_* (*n* = 1–4; *m* = 3–7) [29] and the coordination complexes of telluroethers (see refs. [35,36,37,38,39] for some recent publications). Monotelluroethers Te(CH_2_)*_m_* are liquid in ambient conditions, but species with higher Te contents are solid. The molecular structures and the packing of seven macrocyclic aliphatic telluroethers have been explored [29]. Te(CH_2_)_6_ was one of the monotelluroethers that was synthesized and characterized through NMR spectroscopy, but since it is a liquid at room temperature with a low melting point, its crystal structure determination could not be carried out. It was thought that the reaction with [PtCl_2_(NCPh)_2_] should enable it to coordinate with the platinum center, and the resulting complex would be crystalline. Its crystal structure determination would establish the molecular structure of Te(CH_2_)_6_. The monodentate also ligand avoids the formation of coordination polymers. It turned out that the reaction produced not only the expected *cis*- and *trans*-[PtCl_2_{Te(CH_2_)_6_}_2_] (**1*_cis_*** and **1*_trans_***, respectively), but small amounts of *cis-trans*-[Pt_3_Cl_6_{Te(CH_2_)_6_}_4_]·1¼CH_2_Cl_2_) (**2**·1¼CH_2_Cl_2_) and, depending on the molar ratio of the reagents, also *cis*-*trans*-[Pt_4_Cl_8_{Te(CH_2_)_6_}_4_]·4CDCl_3_ (**3**·4CDCl_3_). The attempts to produce the polynuclear complexes in better yields involved the use of *d*_6_-dimethyl sulfoxide as the crystallization solvent, in which case crystals of the mononuclear [PtCl_2_{S(O)(CD_3_)_2_}{Te(CH_2_)_6_}] (**4**) were obtained. The crystal structures of **1*_cis_*** and***2***-**4**, the isomerization of **1*_cis_*** to **1*_trans_***, and the bonding features and formation of **2** and **3** are discussed in this paper.

## 2. Results and Discussion

### 2.1. General

**Scheme 1:**
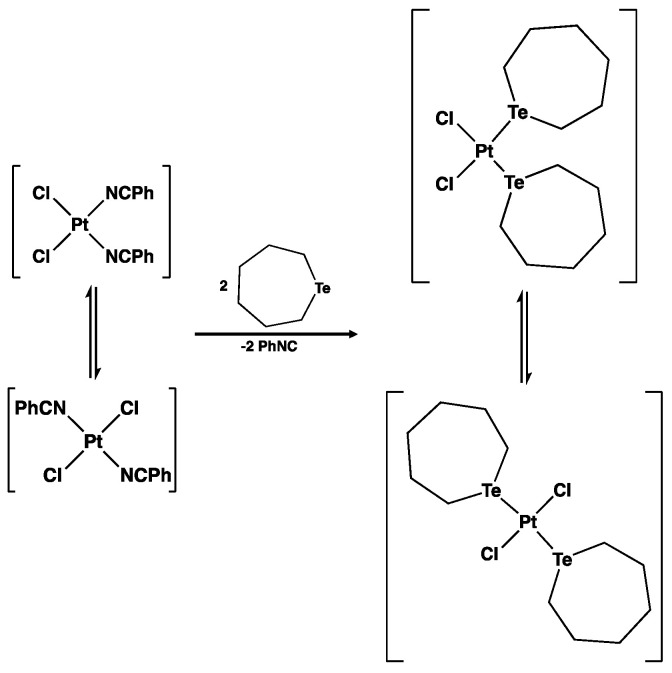
Formation and isomerization of [PtCl_2_{Te(CH_2_)_6_}_2_].

The reaction of two equivalents of Te(CH_2_)_6_ with one equivalent of *cis*-[PtCl_2_(NCPh)_2_] in dichloromethane produces *cis*- and *trans*-[PtCl_2_{Te(CH_2_)_6_}_2_] (**1***_cis_* and **1*_trans_***) (see Scheme 1). The ^1^H, ^125^Te{^1^H}, and ^195^Pt{^1^H} NMR spectra recorded in CDCl_3_ indicate that the *trans*-isomer is the main isomer in CDCl_3_, as discussed in Section 2.2. Small amounts of insoluble, likely polymeric products were also formed. 

The syntheses of platinum and palladium complexes using related telluracyclopentane Te(CH_2_)_4_ have been reported to give [MX_2_{Te(CH_2_)_4_}_2_] (M = Pt, Pd; X = Cl, Br, I) [30]. The [PtCl_2_{Te(CH_2_)_4_}_2_] was deduced to be the *cis* isomer in the solid state on the basis of IR spectroscopy, but the complex was reported to exist as a mixture of *cis*- and *trans*-isomers in solution in the respective concentration ratio of, ca. 1:2. The palladium complex [PdCl_2_{Te(CH_2_)_4_}_2_] exists only as a *trans* isomer [30]. 

The crystallization of the product of the reaction of Te(CH_2_)_6_ and *cis*-[PtCl_2_(NCPh)_2_] from dichloromethane/pentane gave a mixture of yellow plates and orange-red needles. The yellow plate-shaped crystals were suitable for the determination of the crystal structure, which showed them to be *cis*-[PtCl_2_{Te(CH_2_)_6_}_2_] (**1*_cis_***) (see Section 2.3). The orange-red needles were intergrown and proved to be unsuitable for X-ray structure analysis. However, the NMR and mass spectroscopic information indicated them to be *trans*-[PtCl_2_{Te(CH_2_)_6_}_2_] (**1*_trans_***). 

Small amounts of both yellow and orange-red crystals were manually separated under the microscope. They were dissolved in CDCl_3_ for the recording of the NMR spectra (see Section 2.2). An additional small crop of red plate-shaped crystals was identified under the microscope and could be manually isolated based on their different crystal habits. The crystal structure was determined as *cis-trans*-[Pt_3_Cl_6_{Te(CH_2_)_6_}_4_]∙1¼CH_2_Cl_2_ (**2**·1¼CH_2_Cl_2_) (see Section 2.3). Because of the very small amount of **2**, no bulk analysis could be carried out.

The synthesis was repeated by using equimolar amounts of *cis*-[PtCl_2_(NCPh)_2_] and Te(CH_2_)_6_. Thin-layer chromatography indicated mostly the formation of *cis*- and *trans*-[PtCl_2_{Te(CH_2_)_6_}_2_] (**1*_cis_*** and **1*_trans_***) together with the presence of the starting materials. After recording the NMR spectra in CDCl_3_, red well-shaped crystals grew in the NMR tube. The crystal structure determination showed these crystals to be *cis*-*trans*-[Pt_4_Cl_8_{Te(CH_2_)_6_}_4_]∙4CDCl_3_ (**3**∙4CDCl_3_) (see Section 2.3).

During the attempts to crystallize **3** in larger amounts, the recrystallization in dimethyl sulfoxide was attempted. It yielded almost colorless crystals of [PtCl_2_{S(O)(CD_3_)_2_}{Te(CH_2_)_6_}] (**4**) (see Section 2.3).

### 2.2. NMR Spectroscopy

**Figure 1:**
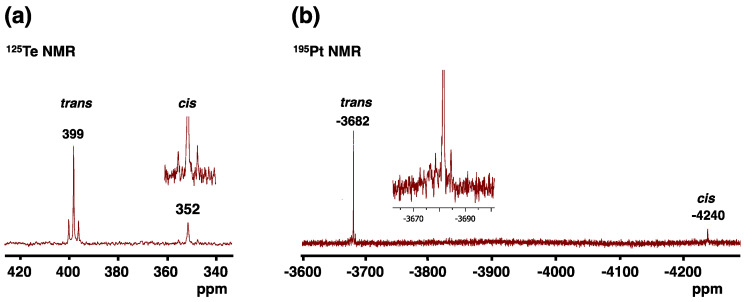
(**a**) The ^125^Te{^1^H} and (**b**) the ^195^Pt{^1^H} NMR spectra of the mixture of the *cis*- and *trans*-isomers of [PtCl_2_{Te(CH_2_)_6_}_2_].

The crystals of *cis*-[PtCl_2_{Te(CH_2_)_6_}_2_] were dissolved in deuterochloroform for recording the ^125^Te{^1^H} NMR and ^195^Pt{^1^H} NMR spectra (see Figure 1). All spectra can be interpreted in terms of a mixture of **1*_cis_*** and **1*_trans_***. The NMR spectroscopic information of **1*_cis_*** and **1*_trans_*** is compared with those of related species in Table 1.

Based on the trends in the ^125^Te and ^195^Pt chemical shifts that have been reported previously [30,40,41], the tellurium resonance at 352 ppm has been assigned to the *cis* isomer **1*_cis_*** and that at 399 ppm to the *trans* isomer **1*_trans_***. It can also be seen in Table 1 that the magnitudes of the ^1^J_TePt_ coupling constants in the *cis*-isomers are almost double compared to those of the corresponding *trans*-isomers. These relative values, as well as the trends in both the ^125^Te and ^195^Pt chemical shifts, reflect the stronger *trans*-influence of the tellurium donors compared to that of the chlorido ligand and support the assignments. 

The *cis* -> *trans* isomerization of [PtCl_2_{Te(CH_2_)_6_}_2_] was monitored using ^1^H NMR spectroscopy (see Figure S1 in Supplementary Materials). The crop of crystals of **1*_cis_*** from which the crystal structure determination was carried out (Section 2.3) was dissolved in CDCl_3_, and the spectra were recorded at room temperature for 30 s after the dissolution and again after 1.2 h. In solution, the *trans*-isomer **1*_trans_*** quickly became the dominant species. Already in the first spectrum (Figure S1a) recorded almost immediately after the dissolution of the crystals, the *trans*-isomer can be observed, and in the second spectrum (Figure S1b), it is clearly the predominant species. The assignment of the ^1^H chemical shifts was verified by recording the ^1^H NMR spectrum of the redissolved orange needles. Immediately after the dissolution, the resonances marked as *trans* in Figure S1 were the major signals in the spectrum and remained so throughout prolonged monitoring of the solution. 

After 1.2 h, the *cis*:*trans* ratio was estimated from the integrated intensities of the resonances to be 1:1.8, which is very close to the ratio of 1:2 that Kemmitt et al. [30] estimated for the related [PtCl_2_{Te(CH_2_)_4_}_2_] in dichloromethane. The intensity distribution in the ^125^Te{^1^H} and ^195^Pt{^1^H} NMR spectra (see Figure 1) bears semiquantitative agreement with the inferences from the ^1^H spectrum in Figure S1b. 

The ^125^Te{^1^H} NMR spectrum of the solidified and redissolved reaction mixture prior to separation of the products is shown in Figure S3 of Supplementary Materials. In addition to the resonances of **1*_cis_*** and **1*_trans_***, two weak signals at 489 ppm and 598 ppm were observed. The qualitative comparison of their relative signal intensities and the chemical shifts might indicate the presence of the trinuclear complex *cis-trans*-[Pt_3_Cl_6_{Te(CH_2_)_6_}_4_] (**2**), but this assignment remains tentative at best. The former resonance could be due to the terminal Te(CH_2_)_6_ ligand, and the latter due to the bridging ligand (see Section 2.3).

### 2.3. Crystal and Molecular Structures

**Figure 2:**
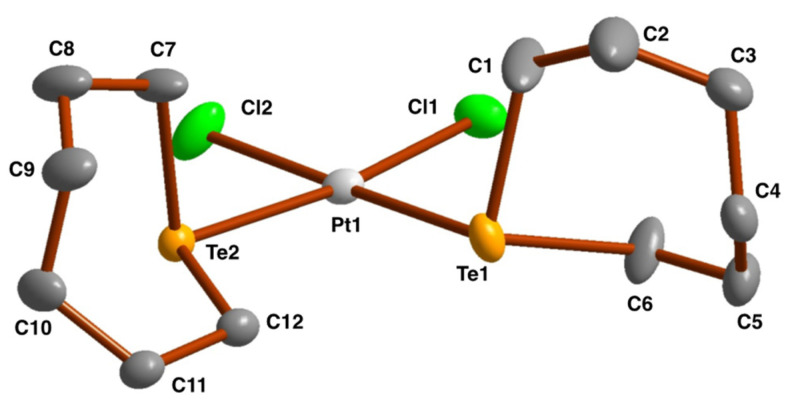
The molecular structure of *cis*-[PtCl_2_{Te(CH_2_)_6_}_2_] (**1*_cis_***) indicating the numbering of the atoms. The thermal ellipsoids have been drawn at the 50% probability level. Hydrogen atoms have been omitted for clarity. Selected bond lengths (Å) and angles (°): Pt1-Te1 2.5145(7), Pt1-Te2 2.5266(6), Pt1-Cl1 2.340(2), Pt1-Cl2 2.359(2), Te1-C1 2.164(10), Te1-C6 2.129(9), Te2-C7 2.166(9), Te2-C12 2.138(8); Cl1-Pt1-Cl2 90.98(9), Cl1-Pt1-Te1 92.67(7), Cl1-Pt1-Te2 172.18(6), Cl2-Pt1-Te1 174.75(6), Cl2-Pt1-Te2 172.18(6), Te1-Pt1-Te2 94.01(2).

Upon slow recrystallization from CH_2_Cl_2_/pentane (1:1) solution, intense yellow plate-shaped crystals and orange needles were obtained in addition to small crops of other products. The yellow plate-shaped crystals were suitable for the determination of the crystal structure and were shown to be *cis*-[PtCl_2_{Te(CH_2_)_6_}_2_] (**1*_cis_***). The details of the data collection and structure determination are presented in Table S1 in Supplementary Materials. The molecular structure and the numbering of the atoms in **1*_cis_*** together with some selected bond parameters are shown in Figure 2. 

The platinum atom exhibits a slightly distorted square planar coordination geometry with all bond parameters showing their expected values (c.f.*,* Pt-Te 2.4971(14)–2.541(14) Å; Pt-Cl 2.311(6)–2.356(6) Å [31,41,42,43]). Some *trans*-[PtCl_2_(TeR_2_)_2_] show longer Pt-Te bond lengths but slightly shorter Pt-Cl bond distances (Pt-Te 2.5589(12)–2.5945(3) Å; Pt-Cl 2.275(5)–2.320(5) Å [31,41,43,44,45]). This is due to the relative strengths of the *trans*-influence of the telluroether and chlorido ligands. 

**Figure 3:**
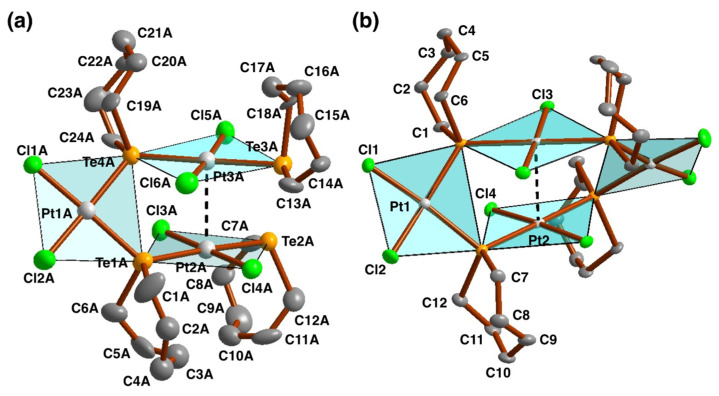
(**a**) Molecular structure of *cis-trans*-[Pt_3_Cl_6_{Te(CH_2_)_6_}_4_]·1¼CH_2_Cl_2_ (**2**·1¼CH_2_Cl_2_) and (**b**) *cis*-*trans*-[Pt_4_Cl_8_{Te(CH_2_)_6_}_4_]·4CDCl_3_ (**3**·4CDCl_3_) indicating the numbering of the atoms. The thermal ellipsoids have been drawn at the 50% probability level. Some Te(CH_2_)_6_ rings and the solvent molecules in **2**·1¼CH_2_Cl_2_ and **3**·4CDCl_3_ are disordered. For clarity, only one disordered ligand-component is shown in the figure. The hydrogen atoms have also been omitted from both complexes for clarity.

In addition to **1*_cis_*** and **1*_trans_***, a few red, plate-shaped crystals of the trinuclear *cis-trans*-[Pt_3_Cl_6_{Te(CH_2_)_6_}_4_]·1¼CH_2_Cl_2_ (**2**·1¼CH_2_Cl_2_) could be manually separated from the reaction mixture after the crystallization from CH_2_Cl_2_/pentane. The crystals of tetranuclear *cis*-*trans*-[Pt_4_Cl_8_{Te(CH_2_)_6_}_4_]·4CDCl_3_ (**3**·4CDCl_3_) could be obtained upon recrystallization from CDCl_3_ in the NMR tube after the recording of the NMR spectra of the equimolar reaction of the reagents. The molecular structures of **2**·1¼CH_2_Cl_2_ and **3**·4CDCl_3_ are shown in Figure 3 (for details of the structure determination and the list of selected bond parameters, see Tables S1 and S2 in Supplementary Materials).

The asymmetric unit of **2**·1¼CH_2_Cl_2_ contains two independent complexes (denoted **A** and **B**) with virtually the same conformations and bond parameters. There are also 2½ solvent molecules in the asymmetric unit. Some Te(CH_2_)_6_ ligands exhibit orientational disorder. Since the structures of both discrete complexes in the asymmetric unit are closely similar, only complex **A** is shown in Figure 3a. In the case of **3**·4CDCl_3_, the asymmetric unit contains only half of the tetranuclear complex, with the other half being completed through symmetry. The geometry of **3** is closely related to that of **2**, as shown in Figure 3b.

Both complexes **2** and **3** show the simultaneous occurrence of very slightly distorted square-planar *cis*- and *trans*-PtCl_2_Te_2_ coordination environments. In the *cis*-moieties, the Pt-Te bonds range from 2.5045(7) to 2.5226(6) Å and the Pt-Cl bonds range from 2.309(3) to 2.331(5) Å. In the *trans*-PtTe_2_Cl_2_ moieties, the Pt-Te and Pt-Cl range from 2.5546(6) to 2.5774(15) Å and from 2.302(2) to 2.326(6) Å, respectively. The relative magnitudes of these metrical values demonstrate the stronger *trans*-influence of tellurium compared to chlorine. 

**Figure 4:**
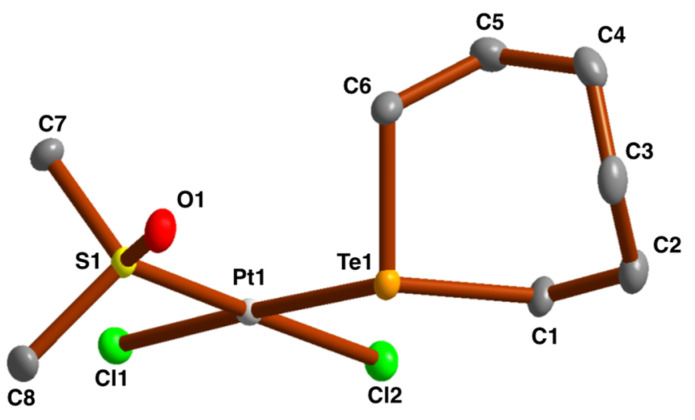
The molecular structure of [PtCl_2_{S(O)(CD_3_)_2_}{Te(CH_2_)_6_}] (**4**) indicating the numbering of the atoms. The thermal ellipsoids have been drawn at the 50% probability level. Hydrogen and deuterium atoms have been omitted for clarity. Selected bond lengths (Å) and angles (°): Pt1-Te1 2.5436(3), Pt1-S1 2.2049(7), Pt1-Cl1 2.3564(7), Pt1-Cl2 2.3203(7), S1-O1 1.479(2), Te1-C1 2.146(3), Te1-C6 2.176(3), Te1-Pt1-Cl1 176.33(2), Te1-Pt1-Cl2 92.32(2), Te1-Pt1-S1 87.68(2), Cl1-Pt1-Cl2 89.78(2), Cl1-Pt1-S1 90.27(3), Cl2-Pt1-S1 179.11(3).

The molecular structure of [PtCl_2_{S(O)(CD_3_)_2_}{Te(CH_2_)_6_}] (**4**) is shown in Figure 4 together with the labeling of atoms and selected bond parameters.

The Pt-Te bond length of 2.5436(3) Å in **4** is in agreement with that of **1*_cis_*** and is consistent with the weak *trans*-influence of the chlorido ligand. The two Pt-Cl bond lengths are 2.3564(7) Å (*trans* to Te) and 2.3203(7) Å (*trans* to S) and reflect also the relative *trans*-influences of tellurium and sulfur. The Pt-S bond length is 2.2049(7) Å. The closest complex related to [PtCl_2_{S(O)(CD_3_)_2_}{Te(CH_2_)_6_}] (**4**) is [PtCl_2_{S(O)(CH_3_)_2_}{S(CH_3_)_2_}] [46] that shows a Pt-S(O)(CH_3_)_2_ distance of 2.211(4) Å. The Pt-Cl bond lengths are 2.299(3) and 2.320(4) Å, and the Pt-S(CH_3_)_2_ distance is 2.294(3) Å. In the Cambridge database, there are nine crystal structure determinations for [PtCl_2_{S(O){CH_3_}_2_}_2_] containing the *trans*-Cl-Pt-S(O)CH_3_ arrangement [47]. The Pt-S bonds span a range of 2.2302(18)–2.2537(12) Å (average 2.242(10) Å), and the Pt-Cl bonds vary in the range of 2.3068(8)–2.3251(5) Å (average 2.317(5) Å). 

**Figure 5:**
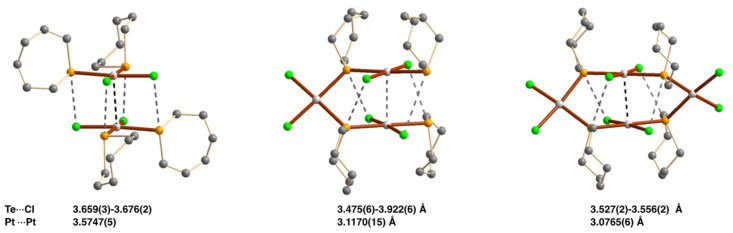
Chalcogen bonds and metallophilic interactions in **1*_cis_***, **2**, and **3**.

In the solid state, all complexes **1*_cis_***, **2**, and **3** show short Te⋯Cl and Pt⋯Pt contacts. These secondary bonding interactions comprise chalcogen bonds (Te⋯Cl) and metallophilic interactions (Pt⋯Pt) that, in the case of **1*_cis_***, link the mononuclear *cis*-[PtCl_2_{Te(CH_2_)_6_}_2_] complexes into dimers exhibiting respective contacts of 3.659(3)–3.676(2) Å and 3.5747(5) Å. While the chalcogen bonds and metallophilic interactions in **2** and **3** are structurally similar to those in **1*_cis_*** (see Table S2), in the case of the latter polynuclear complexes, they are *intra*-molecular (see Figure 5). Similar linking of the square-planar MX_2_E_2_ (M = Pt, Pd; E = Se, Te) coordination spheres into dimers has also been observed in other chalcogenoether complexes provided that the steric bulk of the organic group bonded to the chalcogen atom does not prevent the dimer formation. Typical examples are *cis*-[PdCl_2_{SeMe(C_4_H_3_S)}_2_] [48], *trans*-[PtI_2_(TeMePh)_2_] [49], *cis*-[PdCl_2_{(Te(C_6_H_4_)OMe-1,4)_2_CH_2_}] [50], and *cis*-[PdBr_2_{(TePh)_2_(CH_2_)_3_}] [51]. The QTAIM analysis of these weak secondary bonding interactions is discussed in Section 2.4.4.

**Figure 6:**
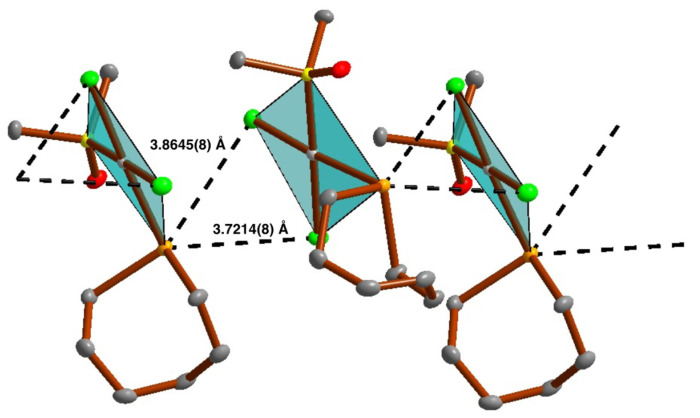
The Te⋯Cl chalcogen bonds in **4**.

Interestingly, [PtCl_2_{S(O)(CD_3_)_2_}{Te(CH_2_)_6_}] (**4**) does not show Pt⋯Pt close contacts (the closest distance is 4.5696(3) Å). The Te⋯Cl contacts of 3.7214(8)–3.8645(8) Å, on the other hand, link the complexes into *quasi*-single-strand polymers (see Figure 6).

### 2.4. Density Functional Theory (DFT) Computations

#### 2.4.1. General 

We carried out PBE0-D3/def2-TZVP calculations to study the bonding, secondary bonding interactions, as well as energetics of the *cis-trans* isomerization of [PtCl_2_{Te(CH_2_)_6_}_2_] and the formation of tri- and tetranuclear complexes. 

#### 2.4.2. Optimized Geometries

The PBE0-D3/def2-TZVP optimized geometries of [PtCl_2_{Te(CH_2_)_6_}_2_] (**1*_cis_*** and **1*_trans_***), *cis-trans*-[Pt_3_Cl_6_{Te(CH_2_)_6_}_4_] (**2**), and *cis*-*trans*-[Pt_4_Cl_8_{Te(CH_2_)_6_}_4_] (**3**) agree well with the experimental information despite the fact that the experimental information is from the crystalline state and the computational data are calculated in vacuum. The PBE0-D3/def2-TZVP-optimized coordinates are shown in Table S3 and the computed geometries are shown in Table S4 in Supplementary Materials. The total energies in vacuum are presented in Table S5 and those in dichloromethane in Table S6. 

#### 2.4.3. *Cis-trans* Isomerization

In vacuum at the PBE0-D3/def2-TZVP level of theory, *trans*-[PtCl_2_{Te(CH_2_)_6_}_2_] (**1*_trans_***) is 33 kJ mol^−1^ more stable than the *cis*-isomer **1*_cis_*** (see Table S5). By contrast, in dichloromethane, the *cis*-isomer is 2 kJ mol^−1^ more stable than the *trans*-isomer (see Table S6). An earlier MP4(SDQ)//MP2 comparison of the Gibbs energies in vacuum and dichloromethane involving *cis*- and *trans*-[PtCl(SnCl_3_)(PH_3_)_2_] (Δ*G*_298_(*cis-trans*) = −32 and 9 kJ mol^−1^, respectively) [52] exhibited the same trend. The computed equilibrium constant for the *cis -> trans* isomerization of [PtCl_2_{Te(CH_2_)_6_}_2_] in dichloromethane utilizing the Gibbs energy change is 0.4. This is consistent with the variable-temperature ^1^H NMR spectroscopic study of the *cis* -> *trans* isomerization of [PdCl_2_(PMePh_2_)_2_] that has been concluded to show a value of 0.30 [53] in 1,2-dichloroethane. In chloroform, *trans*-[PtCl_2_{Te(CH_2_)_6_}_2_] is interestingly 6 kJ mol^−1^ (equilibrium constant 11.2) more stable than the *cis*-form. Since all NMR spectra were recorded in deuterochloroform, this finding explains the observed *cis* -> *trans* isomerization of [PtCl_2_{Te(CH_2_)_6_}_2_] by NMR spectroscopy (see Section 2.2). The route for the *cis* -> *trans* isomerization is generally considered to involve the formation of a five-coordinate intermediate. This intermediate can undergo Berry pseudorotation resulting in the isomerization. Other mechanisms have also been suggested (for early discussion of the possible isomerization mechanisms, see ref. [54]).

#### 2.4.4. Chalcogen Bonding and Metallophilic Interactions

The QTAIM analysis of the secondary bonding Te∙∙∙Cl and Pt∙∙∙Pt interactions are shown in Table 2. The Pt-Te bond lengths and experimental close contacts in the crystals have been included for comparison. The use of QTAIM in the characterization of metal–metal bonding has recently been reviewed [55]. In addition to the classical descriptors of electron density (ρ) and the Laplacian (∇^2^ρ) at the bond critical point that are expected to be large and negative, respectively, for covalent bonds, the relative magnitudes of the kinetic *G*_b_ and the potential *V*_b_ energy densities have been used to classify bonds. The relative magnitudes have been examined either via the electronic energy density (*H*_b_ = *G*_b_ + *V*_b_) [56,57] or the |*V*_b_|/*G*_b_ ratio [58]. The Laplacian for the bonds between heavy atoms tends to give positive values regardless of the bond type [59]. Therefore, the energy densities have been good additional descriptors for defining metal bonding. The negative *H*_b_ values have been taken as a sign of covalent bonding and the positive values as indicators of closed-shell interactions [60]. The |*V*_b_|/*G*_b_ ratio further distinguishes the regions of shared-shell interactions with |*V*_b_|/*G*_b_ > 2, intermediate regions corresponding to 1 < |*V*_b_|/*G*_b_ < 2, and closed-shell interactions with |*V*_b_|/*G*_b_ < 1 [55]. The intermediate region includes metal–metal and donor–acceptor interactions [59].

In the case of complexes **1*_cis_***, **2**, and **3,** the calculated ∇^2^ρ for Pt∙∙∙Pt, Pt–Te, and Te∙∙∙Cl are all positive, as can be expected for interactions between the heavy atoms. For the Pt-Te bonds, the values of *H*_b_ are negative and |*V*_b_|/*G*_b_ ratios are in the intermediate region between 1.64 and 1.75, consistent with the donor–acceptor bonds. The electron densities at bond critical points (𝜌) and the delocalization indices (DIs) that are close to the single bond values [61] corroborate this classification of the coordinative bonds. Because of the *trans*-influence, the Pt-Te bonds are slightly longer than single bonds and cause the delocalization indices of the Pt-Te(*trans*) bonds to be smaller than those of the Pt-Te(*cis*) bonds. By comparison, *H*_b_ of the Pt∙∙∙Pt interactions are still negative but |*V*_b_|/*G*_b_ ratios that fall between 1.06 and 1.15 (see Table 2) are much closer to the limit of closed-shell interactions. The low level of electron sharing in the Pt∙∙∙Pt interactions is reflected by the ρ values 0.122, 0.172, and 0.202 e Å^−3^ and the DI values 0.18, 0.26, and 0.30 for **1*_cis_***, **2**, and **3**, respectively. By comparison, the QTAIM analysis in a recent study on platinum complexes of phenylpyridine, triazolyl-phenylpyridine, and imidazolyl-phenylpyridine that form head-to-tail dimers via the Pt∙∙∙Pt interactions in the solid state show the ρ values of 0.132–0.150 e Å^–3^ that are between those of **1*_cis_*** and **2** [62]. The |*V*_b_|/*G*_b_ ratios of Pt∙∙∙Pt interactions of 1.08–1.10 are also very similar to those found in this contribution. In both cases, the metallophilic Pt∙∙∙Pt interactions show weak covalence.

**Table 2:** QTAIM analysis results (electron density at bond critical point, ρ; delocalization index, DI; Laplacian of electron density, ∇^2^ρ; kinetic energy density, *G_b_*; and potential energy density, *V_b_*) of the Pt⋯Pt and Te⋯Cl secondary bonding interactions in *cis*-[PtCl_2_{Te(CH_2_)_6_}_2_] (**1*_cis_***), *cis-trans*-[Pt_3_Cl_6_{Te(CH_2_)_6_}_4_] (**2**), and *cis*-*trans*-[Pt_4_Cl_8_{Te(CH_2_)_6_}_4_] (**3**).

Parameter	Expl.	PBE0-D3/def2-TZVP	QTAIM					
			ρ (e Å^–3^)	DI	∇^2^ρ (e Å^–5^)	*G_b_* (kJ mol^−1^ bohr^–3^) *^a^*	*V_b_* (kJ mol^−1^ bohr^–3^) *^a^*	*E*_int_ (kJ mol^−1^ bohr^–3^) *^a, b^*
**1** *_cis_*								
Pt-Te(*cis*)	2.5145(7)–2.5266(6)	2.537–2.548	0.619/0.628	0.98/1.03	1.15/1.18	130/133	−228/−234	−114/−117
Pt⋯Pt	3.5747(5)	3.403	0.122	0.18	1.0	29	−31	−16
Te⋯Cl	3.659(3)–3.676(2)	3.516–3.531	0.074–0.068	0.10	0.74–0.67	14	−13–(−14)	−7
**2**								
Pt-Te(*cis*)	2.5140(15)–2.5219(16)	2.515–2.515	0.630–0.634	0.94	1.83–1.85	152–153	−254–(−256)	−127–(−128)
Pt-Te(*trans*)	2.5560(15)–2.5774(15)	2.572–2.581	0.578–0.588	0.81–0.92	1.13–1.77	118–132	−206–(−217)	−103–(−109)
Pt⋯Pt	3.1170(11)–3.1499(13)	3.167	0.172	0.26	1.42	44	−50	−25
Te⋯Cl	3.481(7)–3.964(6)	3.508–3.628	0.074–0.061	0.10–0.07	0.71–0.59	13–17	−11–(−14)	−6–(−7)
**3**								
Pt-Te(*cis*)	2.5043(6)–2.5226(6)	2.510	0.638	0.95	1.88	155	−259	−130
Pt-Te(*trans*)	2.5547(6)–2.5577(6)	2.561	0.595	0.84	1.55	132	−222	−111
Pt⋯Pt	3.0764(6)	3.075	0.202	0.30	1.71	54	−62	−31
Te⋯Cl	3.527(2)–3.556(2)	3.613	0.061	0.06	0.61	14	−11	−6

*^a^* 1 bohr = 0.52918 Å. *^b^ E*_int_ is defined as *V*_b_/2 [63].

The weak Te∙∙∙Cl contacts are classified as closed-shell interactions by the positive *H*_b_ values of 0–3, as shown in Table 2. This is also reflected by the small values of ρ (0.061–0.074 e Å^−3^) and DI (0.06–0.10). They can be compared to the intermolecular Te∙∙∙Te chalcogen bonds in solid macrocyclic telluroethers and are of the same order of magnitude [29]. The relative strengths of the Pt∙∙∙Pt and Te∙∙∙Cl interactions can be qualitatively estimated using *E*_int_ calculated from *V*_b_ [63], although some caution should be exercised when drawing conclusions, as the reliability of the relationship has been questioned [64]. Comparison of the *E*_int_ values shows that in all complexes **1*_cis_***, **2**, and **3**, the metallophilic Pt∙∙∙Pt interactions (*E*_int_ = −16–(−31) kJ mol^–1^ bohr^–3^) are stronger than single Te∙∙∙Cl interactions (*E*_int_ = −6–(−7) kJ mol^–1^ bohr^–3^). The stronger attraction between the Pt centers compared to that between Te and Cl could explain the observation that in all three complexes, the square-planar coordination plane is slightly concave with Pt∙∙∙Pt showing the closest distance between these distorted planes (see Figure S4). However, there are four Te∙∙∙Cl interactions in each complex structure compared to one Pt∙∙∙Pt interaction, suggesting that the total stabilization of the complexes due to Te∙∙∙Cl interactions is on par with the Pt∙∙∙Pt interaction.

#### 2.4.5. Formation of *cis-trans*-[Pt_3_Cl_6_{Te(CH_2_)_6_}_4_] and *cis-trans*-[Pt_4_Cl_8_{Te(CH_2_)_6_}_4_]

**Scheme 2:**
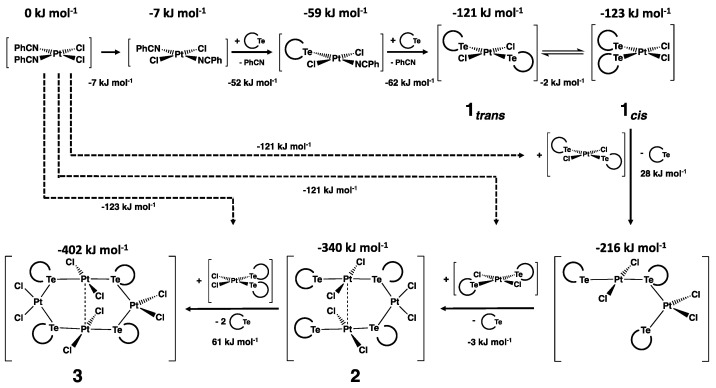
The PBE0-D3/def2-TZVP energetics for the possible route of the formation of *cis*- and *trans*[PtCl_2_{Te(CH_2_)_6_}] (**1*_cis_*** and **1*_trans_***), *cis-trans*-[Pt_3_Cl_6_{Te(CH_2_)_6_}_4_] (**2**), and *cis*-*trans*-[Pt_4_Cl_8_{Te(CH_2_)_6_}_4_] (**3**) in dichloromethane. The relative Gibbs energies of the reaction products and intermediates with respect to the starting complex *cis*-[PtCl_2_)(NCPh)_2_] are shown above each complex, and the Gibbs energy changes in individual transformation steps in connection are shown with the reaction arrows.

The reaction of *cis*-[PtCl_2_(NCPh)_2_] and Te(CH_2_)_6_ afforded small amounts of *cis-trans*-[Pt_3_Cl_6_{Te(CH_2_)_6_}_4_] (**2**) and *cis*-*trans*-[Pt_4_Cl_8_{Te(CH_2_)_6_}_4_] (**3**) that could be identified and structurally characterized through X-ray diffraction. The total PBE0-D3/def2-TZVP energies of all species in dichloromethane (see Table S6) can be used to calculate Gibbs energy changes in the formation of complexes **1*_cis_***, **2**, and **3** from *cis*-[PtCl_2_(NCPh)_2_] and Te(CH_2_)_6_ that are shown in Table S7. One possible route for the formation of the polynuclear complexes is shown in Scheme 2 together with the Gibbs energy changes in the individual reaction steps. The relative Gibbs energies of the reaction products and intermediates with respect to *cis*-[PtCl_2_(NCPh)_2_] are also shown in Scheme 2.

Whereas the energetics in Scheme 2 is favorable for the formation of **2** and **3** (see also Table S6), it seems that only small amounts of these complexes are formed during the syntheses. Though the solid starting material is *cis*-[PtCl_2_(NCPh)_2_], *trans*-[PtCl_2_(NCPh)_2_] is 7 kJ mol^−1^ more stable and therefore, in dichloromethane solution, virtually all *cis*-[PtCl_2_(NCPh)_2_] is converted into the *trans*-isomer. Because, in dichloromethane, *cis*-[PtCl_2_{Te(CH_2_)_6_}_2_] is 2 kJ mol^−1^ more stable than the *trans*-isomer (Table S6), the isomeric composition in equilibrium is, ca., 70% *cis*-[PtCl_2_{Te(CH_2_)_6_}_2_] and 30% *trans*-[PtCl_2_{Te(CH_2_)_6_}_2_]. The *trans*-isomer is therefore the limiting reactant in the formation of **2** and **3**. In fact, the formation of **3** was only observed in the 1:1 reaction of *cis*-[PtCl_2_(NCPh)_2_] and Te(CH_2_)_6_. 

While we have not observed the formation of *cis*-*trans*-[Pt_2_Cl_4_{Te(CH_2_)_6_}_3_] in this reaction, we have previously reported the formation of small amounts of the structurally related *cis-trans*-[Pt_2_Cl_4_{Te(CH_2_SiMe_3_)_2_}_3_] [40].

## 3. Materials and Methods

### 3.1. General Procedures

All manipulations involving air- and moisture-sensitive materials were conducted under a nitrogen atmosphere using Schlenk techniques. Dichloromethane and chloroform were distilled over CaH_2_ and hexane over Na/benzophenone under a nitrogen atmosphere prior to use. Ethanol was degassed by bubbling nitrogen through the solvent for at least 15 min. Semiconductor-grade tellurium was freshly ground. All other reagents were used as purchased without further purification. The preparation of Te(CH_2_)_6_ has been reported earlier [29]. 

### 3.2. Spectroscopy

#### 3.2.1. NMR Spectroscopy

^1^H, ^13^C{^1^H}, ^125^Te{^1^H}, and ^195^Pt{^1^H} NMR spectra of **1*_cis_*** and **1*_trans_*** were recorded in CDCl_3,_ and those of **4** in d*_6_*-DMSO were recorded on a Bruker Avance III 400 spectrometer operating at 400.13, 100.61, 126.24, and 86.02 MHz, respectively. The respective pulse widths were 13.0, 9.70, 6.0, and 10.0 μs, and the corresponding relaxation delay was 2.0 s for each nucleus. The deuterated solvent was used as the ^2^H lock. All resonances were indirectly referenced by using the deuterium signal of the solvent for the lock to the frequency that relates to the resonance frequency of the TMS protons at exactly 400.130000 MHz. Chemical shifts for the ^125^Te resonances are given relative to dimethyl telluride through indirect referencing (the tellurium resonance ν_0_(Te) was calculated by using the ratio Ξ = ν_0,H_(Te)/ν_0_(TMS) = 31.549769% [61]). Chemical shifts for ^195^Pt are given relative to Na_2_[PtCl_6_] (1.2 M in D_2_O) also through indirect referencing (Ξ = ν_0,H_(Pt)*/v*_0_(TMS) = 21.96784%) [65]. The ^1^H and ^13^C are reported relative to tetramethyl silane TMS [66]. 

#### 3.2.2. Mass Spectrometry 

Electron ionization mass spectra were recorded on Finnigan MAT SSQ 710 and Finnigan MAZ95XL spectrometers. The energy of the electrons was 70 eV.

### 3.3. X-ray Diffraction

The crystals of **1**–**4** were coated with Paratone oil and mounted in a nylon CryoLoop, and the intensity data were collected on a Bruker Nonius Kappa CCD diffractometer at 133 K using graphite monochromated Mo Kα radiation (λ = 0.71073 A; 55 kV, 25 mA) [67,68]. Crystal data and the details of structure determinations are given in Table S1. The data were corrected for Lorenz and polarization effects, after which semi-empirical absorption correction was applied to net intensities using SADABS [69]. The structures were solved through direct methods using SHELXT [70] and refined using SHELXL-2018 [71]. After the full-matrix least-squares refinement of the non-hydrogen atoms with anisotropic thermal parameters, the hydrogen atoms were placed in calculated positions in the CH_2_ groups (C-H = 0.99 A). In the final refinement, the hydrogen atoms were riding with the carbon atoms they were bonded to. The isotropic thermal parameters of the hydrogen atoms were fixed at 1.5 times that of the corresponding carbon atoms. The scattering factors for the neutral atoms were those incorporated with the program. 

Some Te(CH_2_)_6_ ligands and solvent molecules in **2**·1¼CH_2_Cl_2_ and **3**·4CDCl_3_ were disordered. The disorder was resolved through appropriate restraining of the anisotropic displacement parameters and some bond lengths. Some parts of the disorder model were introduced by utilizing the program *DSR* [72].

### 3.4. Computational Details

All calculations were performed using the Gaussian 16 program [73] by employing the PBE0 hybrid functional [74,75,76] together with the def2-TZVP basis sets [77,78]. The combination of the PBE0 functional and the def2-TZVP basis set has been shown to be suitable for computational characterization of compounds of heavy p-block elements (see the discussion in ref. [79]). Implicit C-PCM solvent model was applied to treat the solvation effects [80,81], and dispersion forces were treated by using the D3BJ version of Grimme’s empirical correction with Becke–Johnson damping parameterized for the PBE0 functional [82,83,84]. Full structure optimization was carried out for each species considered in this work and the frequencies were calculated for the optimum geometries to ascertain the nature of the stationary points. The quantum theory of atoms in molecules (QTAIM) was used to study *inter*-molecular interactions in the [PtCl_2_{Te(CH_2_)_6_}] (**1*_cis_***, **1*_trans_***), as well as *intra*-molecular interactions in the *cis-trans*-[Pt_3_Cl_6_{Te(CH_2_)_6_}_4_] (**2**) and *cis*-*trans*-[Pt_4_Cl_8_{Te(CH_2_)_6_}_4_] (**3**) structures [85]. AIMAll software was used for the QTAIM calculations [86].

### 3.5. Reaction of Te(CH_2_)_6_ with cis-[PtCl_2_(NCPh)_2_]

Te(CH_2_)_6_ (46.2 mg, 0.218 mmol) was dissolved in 50 mL CH_2_Cl_2_ and crystalline *cis*-[PtCl_2_(NCPh)_2_] (50.0 mg, 0.106 mmol) was added. The mixture was stirred under exclusion of light for 20 h. The solvent was removed under reduced pressure and the crude product was extensively dried at 40 °C and 1 mbar to remove any benzonitrile, yielding 71.7 mg (theoretical: 74.4 mg) of an odorless yellow solid with few orange crystals in it. TLC analysis (silica, chloroform) showed two spots at Rf = 0.89 and 0.25 corresponding to *trans*-[PtCl_2_{Te(CH_2_)_6_}_2_] (**1*_trans_***) and *cis*-[PtCl_2_{Te(CH_2_)_6_}_2_] (**1*_cis_***) beside a very faint spot at Rf = 0.58 and few minor spots at Rf *<* 0.25. The substances corresponding to the latter were mostly removed through column chromatography (silica, chloroform), yielding 54.5 mg of substance. The product was dissolved in CH_2_Cl_2_/pentane and, upon slow evaporation, both **1*_trans_*** and **1*_cis_*** crystallized, with the former giving orange to red, heavily intergrown needles and the latter giving intensely yellow plates suitable for XRD analysis. Most of the substance crystallized in the *cis* form. Small quantities of both isomers could be separated manually. The mixture obtained after the column chromatography still contained a minor side product that could be observed by means of NMR spectroscopy. Therefore, while it is not possible to give exact yields of **1*_trans_*** and **1*_cis_***, the combined overall yield of about 70% can be estimated based on NMR spectroscopy. Few crystals of *cis-trans*-[Pt_3_Cl_6_{Te(CH_2_)_6_}_4_]·1¼CH_2_Cl_2_ (**2**·1¼CH_2_Cl_2_) could also be isolated from the crystalline product mixture due to their different crystal habit. The compound was identified using X-ray diffraction.

*cis*-[PtCl_2_{Te(CH_2_)_6_}_2_] (**1*_cis_***): yellow plates, Rf = 0.25, m.p.: beginning brown coloration at ~140 °C, melting at ~180 °C accompanied by quick decomposition, MS: 690 u/e (M^+^), EA: calcd. C 35.61% H 2.13% N 5.93% Cl 15.02% found C 35.88% H 2.13%, N 6.14% Cl 15.34%. NMR (CDCl_3_): δ_Te_ = 352 ppm, ^1^J_TePt_ = 979 Hz; δ_Pt_ = −4240 ppm.

*trans*-[PtCl_2_{Te(CH_2_)_6_}_2_] (**1*_trans_***): orange to red interwoven needles, Rf = 0.89, m.p.: decomposition at ~150 °C under black coloration, MS: 690 u/e (M^+^). NMR (CDCl_3_): δ_Te_ = 399 ppm, ^1^J_TePt_ = 506 Hz; δ_Pt_ = −3682 ppm.

The reaction was also repeated by using 33.9 mg (0.160 mmol) of Te(CH_2_)_6_ and 75.5 mg (0.160 mmol) of *cis*-[PtCl_2_(NCPh)_2_] in 20 mL of dichloromethane. The reaction progressed in an analogous manner to a 2:1 reaction with **1*_cis_*** and **1*_trans_*** as main products. In addition, the presence of starting materials was observed in the reaction mixture. A few well-shaped red crystals were formed in the NMR tube. They were identified through single-crystal X-ray diffraction as *cis*-*trans*-[Pt_4_Cl_8_{Te(CH_2_)_6_}_4_]·4CDCl_3_ (**3**·4CDCl_3_) (**3**). In order to increase the solubility of the solid material, the recrystallization from dimethyl sulfoxide was attempted. After the NMR measurement in d_6_-dimethyl sulfoxide, pale yellow, almost colorless crystals were formed that, upon the crystal structure determination, proved to be [PtCl_2_{S(O)(CD_3_)_2_}{Te(CH_2_)_6_}] (**4**). NMR (*d*_6_-DMSO): δ_Te_ = 490 ppm, ^1^J_TePt_ = 1052 Hz; δ_Pt_ = −3830 ppm.

## 4. Conclusions

The coordination of Te(CH_2_)_6_ to the Pt(II) center was examined through the reaction of Te(CH_2_)_6_ with [PtCl_2_(NCPh)_2_] (2:1) in dichloromethane. The initial objective was to obtain information about the molecular structure of the Te(CH_2_)_6_ as a ligand in the complex, because the free compound is a thermally unstable and light-sensitive liquid with a low melting point. The main products in the reaction were *cis*- and *trans*-[PtCl_2_{Te(CH_2_)_6_}_2_]. In dichloromethane, the isomers exist as a mixture, but upon crystallization, the *cis* isomer seems to be the dominant species in the solid state. It is likely that in non-polar solvents, the more polar *cis*-isomer is less soluble than the *trans*-isomer. The molecular structures of *cis*-[PtCl_2_{Te(CH_2_)_6_}] and the trinuclear and tetranuclear by-products *cis-trans*-[Pt_3_Cl_6_{Te(CH_2_)_6_}_4_] and *cis*-*trans*-[Pt_4_Cl_8_{Te(CH_2_)_6_}_4_] were determined using X-ray diffraction. These polynuclear complexes show the simultaneous presence of both *cis*-Cl and *trans*-Cl isomers. The secondary bonding interactions involving the Te⋯Cl chalcogen bonds and Pt⋯Pt metallophilic interactions were explored through the use of PBE0-D3/def2-TZVP calculations and discussed using the QTAIM analysis. It turned out that in all complexes, the discrete Pt⋯Pt interaction is stronger than any single Te⋯Cl contact. The total strength of the four Te⋯Cl interactions in each solid lattice is, however, comparable to that of the single Pt⋯Pt interaction. 

*Cis-trans*-[Pt_3_Cl_6_{Te(CH_2_)_6_}_4_] is formed in the 1:2 reaction of *cis*-[PtCl_2_(NCPh)_2_] with Te(CH_2_)_6_, and *cis*-*trans*-[Pt_4_Cl_8_{Te(CH_2_)_6_}_4_] is obtained from the 1:1 reaction. While the overall energetics is favorable to the formation of both complexes, only a few crystals of **2**·1¼CH_2_Cl_2_ were obtained upon crystallization from dichloromethane/pentane. **3**·4CDCl_3_ crystallized on the walls of the NMR tube after the recording of the spectra in both cases. Since the equilibrium composition in dichloromethane contains, ca., 70% of *cis*-[PtCl_2_{Te(CH_2_)_6_}_2_] and 30% of *trans*-[PtCl_2_{Te(CH_2_)_6_}_2_], the latter is the limiting reactant to the formation of tri- and tetranuclear complexes. A significantly larger excess of the *cis*-[PtCl_2_(NCPh)_2_] reagent might improve their yields, but that is the subject for a future study. 

In an attempt to improve the yields of *cis-trans*-[Pt_3_Cl_6_{Te(CH_2_)_6_}_4_] and *cis*-*trans*-[Pt_4_Cl_8_{Te(CH_2_)_6_}_4_], crystallization experiments were performed using dimethyl sulfoxide. This led to the formation of [PtCl_2_{S(O)(CD_3_)_2_}{Te(CH_2_)}] that could be characterized using X-ray diffraction and NMR spectroscopy.

## Figures and Tables

**Table 1:** NMR spectroscopic information of some telluroether complexes of platinum *^a^*.

Compound	δ(^125^Te), ppm		^1^*J*_PtTe_, Hz		δ(^195^Pt), ppm		Ref.
	*cis*	*trans*	*cis*	*trans*	*cis*	*trans*	
[PtCl_2_{Te(CH_2_)_6_}_2_]	352	399	979	506	−4240	−3682	This work
[PtCl_2_{Te(CH_2_)_4_}_2_]	457	459	665	321	−4251	−3715	[30]
[PtCl_2_(TeMe_2_)_2_]	224	234	824	489	−4351	−3765	[40]
[PtCl_2_{Te(CH_2_SiMe_3_)_2_}_2_]	266	292	804	469	−4236	−3707	[41]
[PtCl_2_{S(O)(CD_3_)_2_}{Te(CH_2_)_6_}]	490		1052			−3830	This work

*^a^* The *cis*-designation refers to the relative positions of the two chloride ligands.

## Data Availability

The data presented in this study are openly available in the article.

## Supplementary Materials

The following supporting information can be downloaded at: https://www.mdpi.com/article/10.3390/molecules28227551/s1, Figure S1. ^1^H NMR spectra of the mixture of *cis*- and *trans*-[PtCl_2_{Te(CH_2_)_6_}_2_] (a) 30 s and (b) 1.2 h after the dissolution of the mixture of *cis*-[PtCl_2_{Te(CH_2_)_6_}_2_]. Figure S2. Fluxionality of the Te(CH_2_)_6_ ligand in *cis*- and *trans*-[PtCl_2_{Te(CH_2_)_6_}_2_]. Figure S3. (a) The ^125^Te{^1^H} and (b) the ^195^Pt{^1^H} NMR spectra of the reaction mixture of *cis*-[PtCl_2_(NCPh)_2_] and Te(CH_2_)_6_ after solidification but prior to manual separation of the crystals. Figure S4. The Pt⋯Pt interactions lead the square-planar coordination planes to become concave in **1*_cis_***, **2**, and **3**. Table S1. Crystal data and refinement details for the X-ray structure determinations of *cis*-[PtCl_2_{Te(CH_2_)_6_}_2_] (**1*_cis_***), *cis-trans*-[Pt_3_Cl_6_{Te(CH_2_)_6_}_4_]·1¼CH_2_Cl_2_ (**2**·1¼CH_2_Cl_2_), *cis*-*trans*-[Pt_4_Cl_8_{Te(CH_2_)_6_}_4_]·4CDCl_3_ (**3**·4CDCl_3_), and [PtCl_2_{S(O)(CD_3_)_2_}{Te(CH_2_)_6_}] (**4**). Table S2. Selected bond lengths (Å) and angles (°) of *cis-trans*-[Pt_3_Cl_6_{Te(CH_2_)_6_}_4_]·1¼CH_2_Cl_2_ (**2**·1¼CH_2_Cl_2_) and *cis*-*trans*-[Pt_4_Cl_8_{Te(CH_2_)_6_}_4_]·4CDCl_3_ (**3**·4CDCl_3_). Table S3. Atomic coordinates (Å) of the PBE0-D3/def2-TZVP optimized species discussed in this contribution. Table S4. PBE0-D3/def2-TZVP optimized geometries of the [Pt*_n_*Cl_2*n*_{Te(CH_2_)_6_}*_m_*] (*n* = 1–4; *m* = 2–4). Table S5. Total energies of optimized species at PBE0-D3/def2-TZVP level of theory in vacuum (Hartree). Table S6. Total energies of optimized species at PBE0-D3/def2-TZVP level of theory in dichloromethane (Hartree). Table S7. Gibbs PBE0-D3/def2-TZVP formation energies of **1*_cis_***, **1*_trans_***, **2**, and **3** from *cis*-[PtCl_2_(NCPh)_2_] and Te(CH_2_)_6_ in dichloromethane (kJ mol^–1^). CCDC Deposition Numbers 2298160–2298162 and 2301073 contain the supplementary crystallographic data for this paper. These data are provided free of charge by the joint Cambridge Crystallographic Data Centre and Fachinformationszentrum Karlsruhe Access Structure service www.ccdc.cam.ac.uk/structures.
