# Supplementary material for: Formation, Characterization, and Bonding of cis- and trans-[PtCl2{Te(CH2)6}2], cis-trans-[Pt3Cl6{Te(CH2)6}4], and cis-trans-[Pt4Cl8{Te(CH2)6}4]: Experimental and DFT Study
Source: Molecules. 2023 Nov 12;28(22):7551. doi: 10.3390/molecules28227551 (PMC10673514; doi:10.3390/molecules28227551)
Supplement: Supplementary file 1 [file molecules-28-07551-s001.zip › molecules-2693590-supplementary.pdf]

Formation, Characterization, and Bonding of *cis*- and *trans*-[$PtCl_2\{Te(CH_2)_6\}_2$], *cis-trans*-[$Pt_3Cl_6\{Te(CH_2)_6\}_4$], and *cis-trans*-[$Pt_4Cl_8\{Te(CH_2)_6\}_4$]: Experimental and DFT Study †

Marko Rodewald [1,‡], J. Mikko Rautiainen [2], Helmar Görls [1], Raija Oilunkaniemi [3], Wolfgang Weigand [1,*] and Risto S. Laitinen [3,*]

1 Institute for Inorganic and Analytical Chemistry, Friedrich Schiller University of Jena, Humboldt Str. 8, 07743 Jena, Germany; marko.rodewald@uni-jena.de (M.R.); helmar.goerls@uni-jena.de (H.G.)

2 Department of Chemistry and Nanoscience Center, University of Jyväskylä, P.O. Box 35, 40014 Jyväskylä, Finland; j.mikko.rautiainen@jyu.fi

3 Laboratory of Inorganic Chemistry, Environmental and Chemical Engineering, University of Oulu, P.O. Box 3000, 90014 Oulu, Finland; raija.oilunkaniemi@oulu.fi

* Correspondence: wolfgang.weigand@uni-jena.de (W.W.); risto.laitinen@oulu.fi (R.S.L.)

† Dedicated to Professor J. Derek Woollins on the occasion of his retirement.

‡ Current address: Leibniz Institute of Photonic Technology, Member of Leibniz Health Technologies, Member of the Leibniz Centre for Photonics in Infection Research (LPI), P.O. Box 100239, 07702 Jena, Germany.

Supporting Information

1. NMR spectroscopy

1.1 *^{1}H NMR Spectra of the mixture of cis- and trans-[$PtCl_2\{Te(CH_2)_6\}_2$]*

Figure 1S. ^{1}H NMR spectra of the mixture of *cis*- and *trans*-[$PtCl_2\{Te(CH_2)_6\}_2$] (a) 30 s and (b) 1.2 h after the dissolution of *cis*-[$PtCl_2\{Te(CH_2)_6\}_2$].
Figure 2S. Fluxionality of the $Te(CH_2)_6$ ligand in *cis*- and *trans*-[$PtCl_2\{Te(CH_2)_6\}_2$].

1.2 *$^{125}Te\{^1H\}$ and $^{195}Pt\{^1H\}$ NMR Spectra of the reaction mixture of cis-[$PtCl_2(NCPh)_2$] and $Te(CH_2)_6$*

Figure 3S. (a) The $^{125}Te\{^1H\}$ NMR and (b) the $^{195}Pt\{^1H\}$ NMR spectra from the reaction solution of *cis*-[$PtCl_2(NCPh)_2$] and $Te(CH_2)_6$.

2. X-ray crystallography

Table 1S. Crystal data and refinement details for the X-ray structure determinations of *cis*-[$PtCl_2\{Te(CH_2)_6\}_2$] (**1**$_{cis}$), *cis-trans*-[$Pt_3Cl_6\{Te(CH_2)_6\}_4$] ·1¼CH_2Cl_2 (**2**·1¼CH_2Cl_2), *cis-trans*-[$Pt_4Cl_8\{Te(CH_2)_6\}_4$] ·4$CDCl_3$ (**3**·4$CDCl_3$), and [$PtCl_2\{S(O)(CD_3)_2\}\{Te(CH_2)_6\}$] (**4**).
Table 2S. Selected bond lengths (Å) and angles (°) in *cis-trans*-[$Pt_3Cl_6\{Te(CH_2)_6\}_4$]·1¼CH_2Cl_2 (**2**·1¼CH_2Cl_2) and *cis-trans*-[$Pt_4Cl_8\{Te(CH_2)_6\}_4$]·4$CDCl_3$ (**3**·4$CDCl_3$).

3. DFT Computations

3.1 Optimum geometries

Table 3S. Atomic coordinates (Å) of the PBE0-D3/def2-TZVP optimized species discussed in this contribution.
Table 4S. PBE0-D3/def2-TZVP optimized geometries of the [$Pt_nCl_{2n}\{Te(CH_2)_6\}_m$] ($n$ = 1-4; m = 2-4).
Table 5S. Total energies of optimized species at PBE0-D3/def2-TZVP level of theory in vacuum (Hartree).
Table 6S. Total energies of optimized species at PBE0-D3/def2-TZVP level of theory in dichloromethane (Hartree).

3.2 Secondary bonding interactions

Figure 4S. The Pt···Pt interactions result in the square-planar coordination plane to become slightly concave in (a) $\mathbf{1}_{cis}$, (b) **2**, and (c) **3**.

3.3 Formation energetics

Table 7S. Gibbs formation energies calculated at PBE0-D3/def2-TZVP level of theory (kJ mol^{-1}) in dichloromethane.

1. NMR spectroscopy

1.1 ^{1}H NMR spectra of the mixture of cis- and trans-[PtCl$_2${Te(CH$_2$)$_6$}$_2$]

The ^{1}H spectra of the mixture of *cis*- and *trans*-[PtCl$_2${Te(CH$_2$)$_6$}$_2$] has been shown in Figure 1S both after 30 s and 1.2 h after the dissolution of *cis*-[PtCl$_2${Te(CH$_2$)$_6$}$_2$]. The multiplets were assigned to the *cis*- and *trans*-form based on relative intensities.

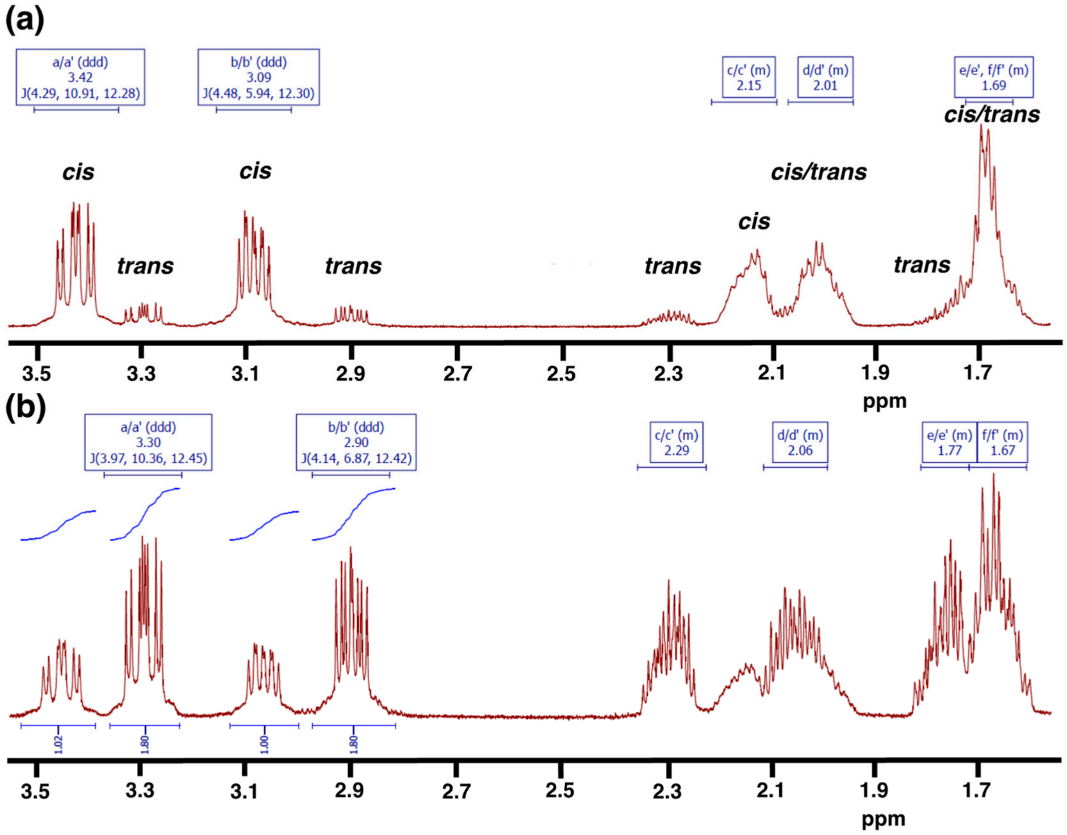

Figure S1. ^{1}H NMR spectra of the mixture of *cis*- and *trans*-[PtCl$_2${Te(CH$_2$)$_6$}$_2$] (a) 30 s and (b) 1.2 h after the dissolution of *cis*-[PtCl$_2${Te(CH$_2$)$_6$}$_2$].

The ^{1}H NMR spectra of both species exhibit very complex multiplets. Since tellurium donates its *n*p lone pair to the Pt-Te bond, the C-Te-C plane will be approximately perpendicular to this bond. The Te(CH$_2$)$_6$ ring is fluxional interconverting between two conformations, appearing planar in the NMR time scale (see Figure 2S). Therefore, the protons on the side of the ring pointing towards the platinum center in Te(CH$_2$)$_6$ aren't chemically equivalent to the ones pointing away from the metal center giving rise to a complicated [AA'BB'CC'DD'EE'FF'] spin system. In case of the free ligand, significantly simpler [ABC]$_4$ spin system is observed [1].

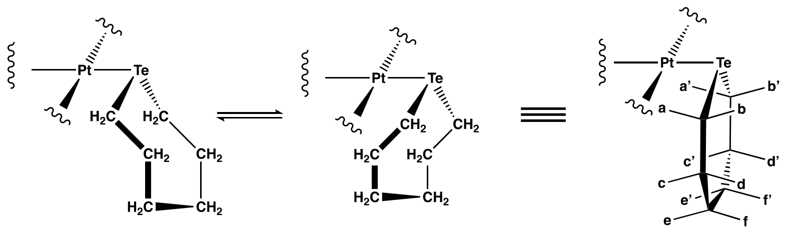

Figure 2S. Fluxionality of the Te(CH$_2$)$_6$ ligand in *cis*- and *trans*-[PtCl$_2${Te(CH$_2$)$_6$}$_2$].

The chemical shifts of the geminal methylene protons closest to tellurium lie at lowest field and are sufficiently different for the second order effects to play only a minor role, which can therefore be ignored in the estimation of coupling constants between the protons a and b (closest to tellurium), and between the protons of position a/b and c/d (see Figure 1S). The geminal coupling constants of $|^2J_{aa'}|$=12.3 Hz (*cis* isomer) and $|^2J_{aa'}|$=12.4 Hz (*trans* isomer) are rather typical for cycloalkane derivatives. The signal assignment to hydrogens **a** and **a'** is not unambiguous but that does not preclude the assignment of these two multiplets to the pair ***a*/a'.** This model is consistent with the slight lowfield shift all α-methylene signals experience compared to the free ligand [1].

It can clearly be seen in Figure 1S(a) that immediately after the dissolution, *cis*-[$PtCl_2\{Te(CH_2)_6\}_2$] is the major species, but the relative concentration of *trans*-[$PtCl_2\{Te(CH_2)_6\}_2$] rapidly increases as a function of time [Figure 1S(b)].

1.2 $^{125}Te\{^1H\}$ and $^{195}Pt\{^1H\}$ NMR spectra of the reaction mixture of cis-[$PtCl_2(NCPh)_2$] and $Te(CH_2)_6$

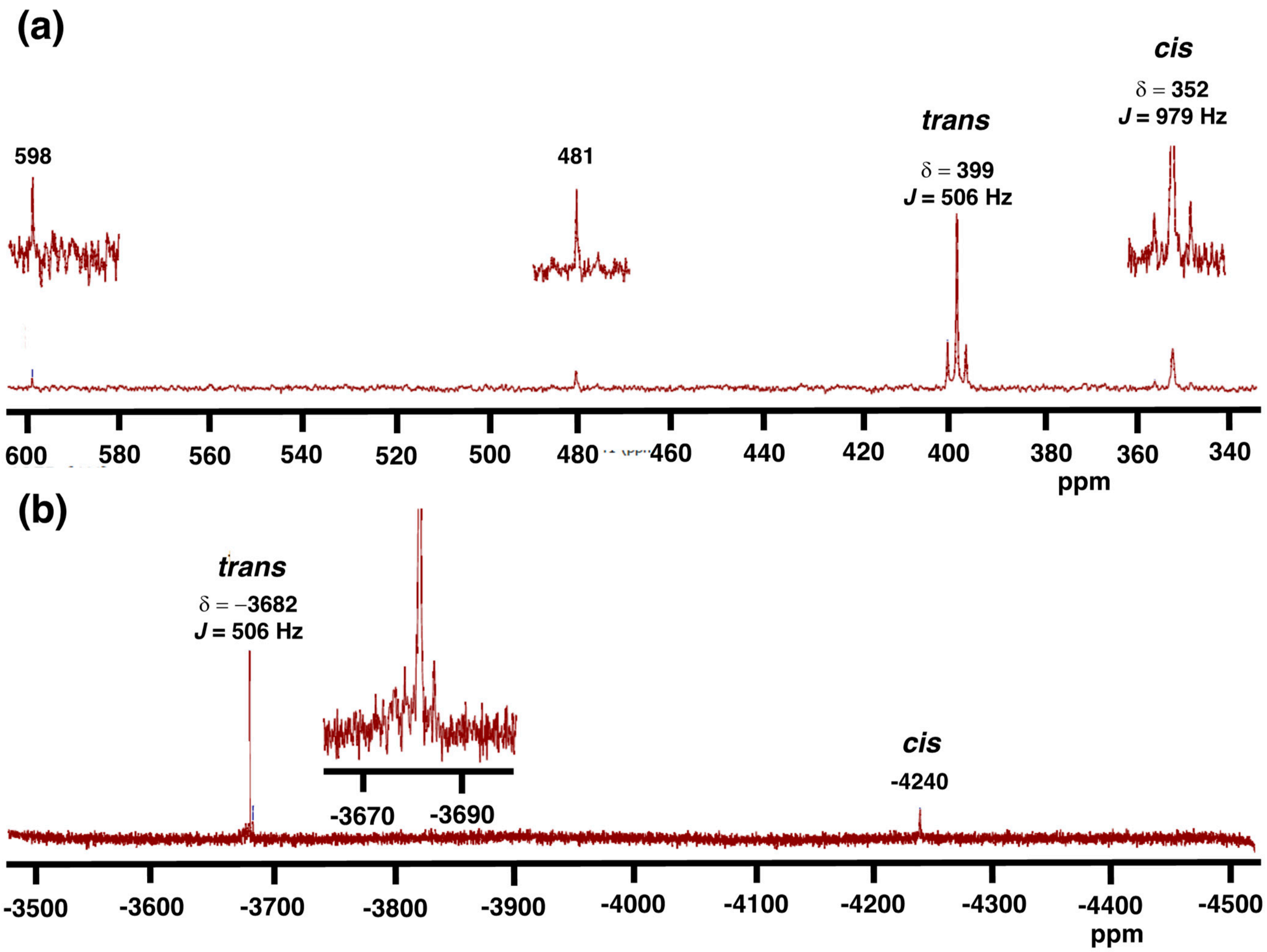

Figure S3. (a) The $^{125}Te\{^1H\}$ NMR and (b) the $^{195}Pt\{^1H\}$ NMR spectra from the reaction solution of *cis*-[$PtCl_2(NCPh)_2$] and $Te(CH_2)_6$.

2. X-ray crystallography

Table S1. Crystal data and refinement details for the X-ray structure determinations of *cis*-$[PtCl_2\{Te(CH_2)_6\}_2]$ (**1**$_{cis}$), *cis-trans*-$[Pt_3Cl_6\{Te(CH_2)_6\}_4]\cdot 1\frac{1}{4}CH_2Cl_2$ (**2**$\cdot 1\frac{1}{4}CH_2Cl_2$), *cis-trans*-$[Pt_4Cl_8\{Te(CH_2)_6\}_4]\cdot 4CDCl_3$ (**3**$\cdot 4CDCl_3$), and $[PtCl_2\{S(O)(CD_3)_2\}\{Te(CH_2)_6\}]$ (**4**).

Compound	**1**$_{cis}$	**2**$\cdot 1\frac{1}{4}CH_2Cl_2$	**3**$\cdot 4CDCl_3$ [a]	**4** [a]
formula	$C_{12}H_{24}Cl_2PtTe_2$	$C_{25.5}H_{51}Cl_9Pt_3Te_4$	$C_{28}H_{52}Cl_{20}Pt_4Te_4$	$C_8H_{18}Cl_2OPtS\ Te$
fw ($g\cdot mol^{-1}$)	689.50	1772.38	2388.45	555.87
T/°*C*	-140(2)	-140(2)	-140(2)	-140(2)
crystal system	orthorhombic	triclinic	monoclinic	monoclinic
space group	*Pbca*	$P\bar{1}$	*C*2/*c*	$P2_1/c$
a/ Å	12.4443(3)	12.5826(4)	12.7358(3)	9.9274(2)
b/ Å	12.8290(3)	12.6959(4)	18.6821(5)	16.3202(4)
c/ Å	21.2611(4)	26.7884(8)	24.5098(6)	9.0309(2)
α/°	90	93.341(1)	90	90
β/°	90	98.757(1)	97.857(1)	108.8660(10)
γ/°	90	90.571(1)	90	90
$V/Å^3$	3394.29(13)	4221.5(2)	5776.9(3)	1384.56(5)
Z	8	4	4	4
ρ ($g\cdot cm^{-3}$)	2.699	2.789	2.746	2.667
μ (cm^{-1})	119.33	132.15	125.85	127.07
measured data	19066	35285	32246	15959
data with $I > 2\sigma(I)$	3337	14448	6312	3092
unique data (R_{int})	3887/0.0784	18271/0.0417	6614/0.0381	3167/0.0263
wR_2 (all data, on F^2) [b]	0.0825	0.2461	0.0939	0.0339
R_1 ($I > 2\sigma(I)$) [b]	0.0433	0.0787	0.0411	0.0150
S [c]	1.093	1.067	1.114	1.119
Res. Dens./$e\cdot Å^{-3}$	1.993/-1.591	6.215/-1.735	5.661/-2.911	0.612/-0.903
absorpt method	multi-scan	multi-scan	multi-scan	multi-scan
absorpt corr $T_{min}/_{max}$	0.3791/0.7456	0.2703/0.7456	0.4244/0.7456	0.5337/0.7456
CCDC No.	2298160	2298161	2298162	2301073

[a] D atoms have been designated as H. [b] Definition of the *R* indices: $R_1 = (\Sigma || F_o| - |F_c||)/\Sigma |F_o|$; $wR_2 = \{\Sigma[w(F_o^2-F_c^2)^2]/\Sigma[w(F_o^2)^2]\}^{1/2}$ with $w^{-1} = {}^2(F_o^2) + (aP)^2 + bP$; $P = [2F_c^2 + Max(F_O^2]/3$; [c] $S = \{\Sigma[w(F_o^2-F_c^2)^2]/(N_o-p..)\}^{1/2}$.

Table S2. Selected bond lengths (Å) and angles (°) in *cis-trans*-$[Pt_3Cl_6\{Te(CH_2)_6\}_4]\cdot 1¼CH_2Cl_2$ (**2**$\cdot 1¼CH_2Cl_2$), and *cis-trans*-$[Pt_4Cl_8\{Te(CH_2)_6\}_4]\cdot 4CDCl_3$ (**3**$\cdot 4CDCl_3$).

	**2**$\cdot 1¼CH_2Cl_2$						**3**$\cdot 4CDCl_3$		
	A [a]	B [a]		A [a]	B [a]				
Pt1-Te1	2.5178(15)	2.5140(15)	Te1-Pt1-Te4	92.26(5)	92.13(5)	Pt1-Te1	2.5045(7)	Te1-Pt1-Te2	94.86(2)
Pt1-Te4	2.5170(16)	2.5219(16)	Te1-Pt1-Cl1	178.57(13)	178.57(14)	Pt1-Te2	2.5226(6)	Te1-Pt1-Cl1	88.16(6)
Pt2-Te1	2.5774(15)	2.5768(17)	Te1-Pt1-Cl2	88.88(13)	88.249(2)	Pt2-Te1	2.5577(6)	Te1-Pt1-Cl2	175.20(7)
Pt2-Te2	2.5560(15)	2.568(2)	Te4-Pt1-Cl1	89.16(14)	89.35(14)	Pt2-Te2	2.5546(6)	Te2-Pt1-Cl1	173.72(7)
Pt3-Te3	2.5608(17)	2.5626(15)	Te4-Pt1-Cl2	177.75(14)	178.45(15)	Pt1-Cl1	2.321(2)	Te2-Pt1-Cl2	87.06(7)
Pt3-Te4	2.5640(15)	2.5635(14)	Cl1-Pt1-Cl2	88.88(14)	88.28(15)	Pt1-Cl2	2.309(3)	Cl1-Pt1-Cl2	90.35(9)
Pt1-Cl1	2.331(5)	2.330(5)	Te1-Pt2-Te2	176.56(5)	173.87(6)	Pt2-Cl3	2.305(2)	Te1-Pt2-Te2	178.92(2)
Pt1-Cl2	2.311(6)	2.328(6)	Te1-Pt2-Cl3	84.33(13)	83.57(13)	Pt2-Cl4	2.302(2)	Te1-Pt2-Cl3	93.43(6)
Pt2-Cl3	2.307(5)	2.313(5)	Te1-Pt2-Cl4	95.79(14)	94.97(13)	Pt2···Pt2	3.0765(6)	Te1-Pt2-Cl4	86.29(6)
Pt2-Cl4	2.305(6)	2.316(5)	Te2-Pt2-Cl3	94.74(13)	94.32(13)	Te1···Cl3	3.527(2)	Te2-Pt2-Cl3	85.52(6)
Pt3-Cl5	2.309(5)	2.325(6)	Te2-Pt2-Cl4	85.16(13)	87.29(13)	Te2···Cl4	3.556(2)	Te2-Pt2-Cl4	94.77(6)
Pt3-Cl6	2.313(6)	2.320(6)	Cl3-Pt2-Cl4	179.8(2)	178.3(2)			Cl3-Pt2-Cl4	175.77(7)
Pt2···Pt3	3.1499(13)	3.1170(11)	Te3-Pt3-Te4	178.14(6)	179.28(5)				
Te1···Cl6	3.481(7)	3.512(5)	Te3-Pt3-Cl5	85.47(14)	86.53(15)				
Te2···Cl5	3.922(6)	3.964(6)	Te3-Pt3-Cl6	95.62(16)	95.26(13)				
Te3···Cl4	3.594(6)	3.435(6)	Te4-Pt3-Cl5	92.72(14)	92.78(15)				
Te4···Cl3	3.590(5)	3.590(5)	Te4-Pt3-Cl6	86.16(16)	85.44(13)				
			Cl5-Pt3-Cl6	174.70(14)	175.3(2)				

[a] The asymmetric unit contains two independent molecules that have been denoted by A and B.

3. DFT Computations[1]

3.1 Optimum geometries

Table S3. Atomic coordinates (Å) of the PBE0-D3/def2-TZVP optimized species discussed in this contribution.

PhCN C_{2v} E=-324.228470092 a.u.

C	0.000000000	0.000000000	0.604835000	H	0.000000000	2.141688000	-2.014339000
C	0.000000000	1.209525000	-0.089890000	H	0.000000000	0.000000000	-3.248749000
C	0.000000000	1.203159000	-1.473355000	H	0.000000000	-2.141688000	-2.014339000
C	0.000000000	0.000000000	-2.165113000	H	0.000000000	-2.141617000	0.461061000
C	0.000000000	-1.203159000	-1.473355000	C	0.000000000	0.000000000	2.031910000
C	0.000000000	-1.209525000	-0.089890000	N	0.000000000	0.000000000	3.183492000
H	0.000000000	2.141617000	0.461061000				

Te(CH$_2$)$_6$ C_1 E=-503.704254999 a.u.

Te	-1.210147000	-0.092563000	-0.101255000	C	2.574441000	-0.570581000	-0.086381000
C	-0.142466000	1.767622000	0.146456000	H	3.411818000	-0.895108000	-0.712723000
H	-0.249894000	2.288014000	-0.807522000	H	2.966064000	-0.528994000	0.937879000
H	-0.672345000	2.335706000	0.910062000	C	1.501412000	-1.646643000	-0.166658000
C	1.320721000	1.610532000	0.506850000	H	1.189195000	-1.776675000	-1.207944000
H	1.411655000	1.145454000	1.493685000	H	1.962004000	-2.595596000	0.137101000
H	1.726403000	2.622252000	0.625499000	C	0.273519000	-1.425347000	0.698719000
C	2.172907000	0.840225000	-0.509266000	H	0.529626000	-1.090026000	1.704770000
H	1.652078000	0.801754000	-1.472985000	H	-0.295924000	-2.351006000	0.806406000
H	3.093740000	1.402635000	-0.687287000				

cis-[PtCl$_2$(NCPh)$_2$] C_s E=-1688.06794514 a.u.

C	*-1.448801000*	*-3.655410000*	*0.000000000*	*Cl*	*2.748639000*	*1.625916000*	*0.000000000*
C	*-2.843133000*	*-3.708134000*	*0.000000000*	*N*	*-0.148166000*	*1.446649000*	*0.000000000*
C	*-3.472666000*	*-4.938992000*	*0.000000000*	*C*	*-0.765694000*	*2.413804000*	*0.000000000*
C	*-2.721177000*	*-6.106566000*	*0.000000000*	*C*	*-1.448598000*	*3.655540000*	*0.000000000*
C	*-1.333906000*	*-6.049577000*	*0.000000000*	*C*	*-2.842946000*	*3.708161000*	*0.000000000*
C	*-0.687992000*	*-4.827309000*	*0.000000000*	*C*	*-0.687892000*	*4.827497000*	*0.000000000*
H	*-3.417083000*	*-2.790020000*	*0.000000000*	*C*	*-3.472576000*	*4.938963000*	*0.000000000*
H	*-4.554686000*	*-4.988661000*	*0.000000000*	*H*	*-3.416816000*	*2.789996000*	*0.000000000*
H	*-3.220818000*	*-7.068098000*	*0.000000000*	*C*	*-1.333906000*	*6.049717000*	*0.000000000*
H	*-0.751248000*	*-6.962619000*	*0.000000000*	*H*	*0.393210000*	*4.759767000*	*0.000000000*
H	*0.393104000*	*-4.759480000*	*0.000000000*	*C*	*-2.721176000*	*6.106599000*	*0.000000000*
C	*-0.765938000*	*-2.413644000*	*0.000000000*	*H*	*-4.554600000*	*4.988549000*	*0.000000000*
N	*-0.148356000*	*-1.446524000*	*0.000000000*	*H*	*-0.751316000*	*6.962802000*	*0.000000000*
Pt	*1.166735000*	*-0.000007000*	*0.000000000*	*H*	*-3.220893000*	*7.068092000*	*0.000000000*
Cl	*2.748412000*	*-1.626185000*	*0.000000000*				

trans-[PtCl$_2$(NCPh)$_2$] D_{2h} E=-1688.07709399 a.u.

C	0.000000000	0.000000000	4.484879000	C	0.000000000	0.000000000	3.068206000
C	0.000000000	1.214614000	5.173137000	N	0.000000000	0.000000000	1.922951000
C	0.000000000	1.205326000	6.555534000	Pt	0.000000000	0.000000000	0.000000000
C	0.000000000	0.000000000	7.244835000	Cl	0.000000000	-2.307595000	0.000000000
C	0.000000000	-1.205326000	6.555534000	Cl	0.000000000	2.307595000	0.000000000
C	0.000000000	-1.214614000	5.173137000	N	0.000000000	0.000000000	-1.922951000
H	0.000000000	2.144470000	4.618226000	C	0.000000000	0.000000000	-3.068206000
H	0.000000000	2.142823000	7.097943000	C	0.000000000	0.000000000	-4.484879000
H	0.000000000	0.000000000	8.328483000	C	0.000000000	-1.214614000	-5.173137000
H	0.000000000	-2.142823000	7.097943000	C	0.000000000	1.214614000	-5.173137000
H	0.000000000	-2.144470000	4.618226000	C	0.000000000	-1.205326000	-6.555534000

H	0.000000000	-2.144470000	-4.618226000
C	0.000000000	1.205326000	-6.555534000
H	0.000000000	2.144470000	-4.618226000
C	0.000000000	0.000000000	-7.244835000
H	0.000000000	-2.142823000	-7.097943000
H	0.000000000	2.142823000	-7.097943000
H	0.000000000	0.000000000	-8.328483000

***cis*-[PtCl$_2${Te(CH$_2$)$_6$}$_2$] C_1 E=-2047.06703240 a.u.**

Pt	-0.146560000	-1.095428000	0.099196000
Te	1.532599000	0.680053000	-0.550223000
Te	-2.219050000	-0.074417000	-0.933730000
Cl	-1.724300000	-2.744233000	0.613361000
Cl	1.557618000	-2.281023000	1.147292000
C	2.318033000	1.174401000	1.378314000
H	1.462031000	1.550590000	1.939703000
H	2.576882000	0.203201000	1.797820000
C	3.464858000	2.172227000	1.332542000
H	4.025965000	2.079756000	2.270148000
H	3.054136000	3.186251000	1.322355000
C	4.421598000	2.047322000	0.153133000
H	3.863665000	2.241474000	-0.772732000
H	5.148153000	2.862974000	0.219700000
C	5.192048000	0.740076000	0.012122000
H	5.845451000	0.609768000	0.881429000
H	5.859166000	0.849909000	-0.850067000
C	4.372891000	-0.536219000	-0.164548000
H	5.052469000	-1.331090000	-0.493132000
H	3.967926000	-0.891236000	0.783303000
C	3.252532000	-0.469616000	-1.182676000
H	2.839265000	-1.458772000	-1.370607000
H	3.569919000	-0.015892000	-2.124201000
C	-1.805695000	2.019445000	-1.171011000
H	-1.004865000	2.017458000	-1.914054000
H	-2.684170000	2.458469000	-1.648274000
C	-1.401339000	2.790625000	0.074782000
H	-0.815591000	2.151988000	0.746843000
H	-0.729820000	3.597640000	-0.239314000
C	-2.557514000	3.414072000	0.845472000
H	-3.207767000	3.942179000	0.136931000
H	-2.144831000	4.186098000	1.502725000
C	-3.402168000	2.486623000	1.707071000
H	-2.764176000	2.008478000	2.459752000
H	-4.099736000	3.117589000	2.266281000
C	-4.212003000	1.404125000	0.995034000
H	-4.620035000	1.788384000	0.052997000
H	-5.086396000	1.177388000	1.615270000
C	-3.492531000	0.084668000	0.792957000
H	-4.188977000	-0.738043000	0.635485000
H	-2.862215000	-0.181446000	1.639656000

***trans*-[PtCl$_2${Te(CH$_2$)$_6$}$_2$] C_1 E=-2047.08131913 a.u.**

Pt	-0.000041000	-0.741429000	0.000040000
Te	2.532226000	-0.744071000	0.507497000
Te	-2.532314000	-0.744031000	-0.507416000
Cl	-0.231568000	-0.765587000	2.303753000
Cl	0.231469000	-0.765956000	-2.303673000
C	2.657679000	1.086279000	1.621284000
H	2.349058000	0.748860000	2.611282000
H	3.716294000	1.352034000	1.662675000
C	1.774308000	2.223153000	1.144918000
H	1.651074000	2.909937000	1.991249000
H	0.778943000	1.822027000	0.933380000
C	2.275387000	3.029405000	-0.042640000
H	1.553719000	3.835630000	-0.211601000
H	3.217208000	3.524702000	0.226473000
C	2.474635000	2.281258000	-1.356944000
H	2.543436000	3.029510000	-2.151192000
H	1.590285000	1.679013000	-1.590091000
C	3.725693000	1.397534000	-1.417757000
H	4.228643000	1.550030000	-2.379606000
H	4.447951000	1.719635000	-0.660058000
C	3.476059000	-0.092378000	-1.307263000
H	4.405765000	-0.660004000	-1.364909000
H	2.796224000	-0.441359000	-2.086707000
C	-3.476057000	-0.092042000	1.307286000
H	-4.405786000	-0.659616000	1.365060000
H	-2.796200000	-0.440928000	2.086753000
C	-3.725578000	1.397903000	1.417533000
H	-4.447712000	1.719968000	0.659700000
H	-4.228623000	1.550603000	2.379299000
C	-2.474393000	2.281446000	1.356699000
H	-1.590143000	1.679076000	1.589905000
H	-2.543101000	3.029761000	2.150895000
C	-2.274995000	3.029457000	0.042342000
H	-3.216730000	3.524885000	-0.226831000
H	-1.553196000	3.835576000	0.211247000
C	-1.774030000	2.223015000	-1.145136000
H	-0.778723000	1.821770000	-0.933544000
H	-1.650693000	2.909690000	-1.991539000
C	-2.657556000	1.086211000	-1.621384000
H	-2.348972000	0.748639000	-2.611343000
H	-3.716136000	1.352095000	-1.662807000

[Pt$_2$Cl$_4${Te(CH$_2$)$_6$}$_3$] C_1 E=-3590.43986476 a.u.

Pt	2.623285000	1.047304000	0.075026000
Pt	-1.599956000	-0.241632000	-0.400069000
Te	2.249045000	-1.444715000	-0.326524000
Te	-3.473540000	-2.000594000	-0.639439000
Te	0.188951000	1.573352000	-0.140589000
Cl	2.845130000	3.353633000	0.420918000
Cl	4.871370000	0.717947000	0.462798000
Cl	-2.410751000	0.616068000	-2.383382000
Cl	-0.727353000	-1.107002000	1.567083000
C	4.055866000	-2.014874000	-1.322886000

H	4.024304000	-1.483250000	-2.275060000
H	4.853595000	-1.567493000	-0.734271000
C	4.182958000	-3.518191000	-1.506898000
H	5.231884000	-3.736226000	-1.741291000
H	3.604955000	-3.826567000	-2.383324000
C	3.739180000	-4.376090000	-0.328619000
H	2.654025000	-4.266941000	-0.200376000
H	3.885269000	-5.425825000	-0.601331000
C	4.428225000	-4.135458000	1.009867000
H	5.503976000	-4.311034000	0.903126000
H	4.065478000	-4.905553000	1.699403000
C	4.220288000	-2.768649000	1.665093000
H	4.490819000	-2.856728000	2.723526000
H	4.902419000	-2.017500000	1.263098000
C	2.804186000	-2.236366000	1.606210000
H	2.663173000	-1.387634000	2.272366000
H	2.049551000	-2.991355000	1.833644000
C	-5.171637000	-0.689656000	-0.711354000
H	-5.177585000	-0.411215000	-1.765567000
H	-6.045506000	-1.315146000	-0.515905000
C	-5.110505000	0.544654000	0.167806000
H	-5.831592000	1.261782000	-0.242771000
H	-4.126552000	1.007074000	0.049021000
C	-5.426328000	0.356271000	1.642999000
H	-5.383122000	1.346004000	2.109161000
H	-6.465566000	0.021355000	1.752467000
C	-4.523906000	-0.587177000	2.431969000
H	-4.682453000	-0.380681000	3.493586000
H	-3.470772000	-0.356918000	2.238167000
C	-4.779341000	-2.078819000	2.187258000
H	-4.795021000	-2.606133000	3.147811000
H	-5.778977000	-2.221256000	1.763660000
C	-3.745224000	-2.788661000	1.338471000
H	-3.996630000	-3.840211000	1.193942000
H	-2.749400000	-2.718446000	1.779210000
C	-0.078362000	3.171994000	-1.523538000
H	0.939572000	3.422008000	-1.820214000
H	-0.623595000	2.712868000	-2.349636000
C	-0.813571000	4.354949000	-0.932956000
H	-0.193537000	4.820693000	-0.161824000
H	-0.883907000	5.093571000	-1.738910000
C	-2.219027000	4.060494000	-0.390459000
H	-2.594927000	3.123719000	-0.818727000
H	-2.895947000	4.838519000	-0.752326000
C	-2.337022000	4.030385000	1.131886000
H	-1.832752000	4.913643000	1.541770000
H	-3.393863000	4.138803000	1.396197000
C	-1.822320000	2.787232000	1.842064000
H	-2.393521000	1.908877000	1.520923000
H	-2.024014000	2.898232000	2.913681000
C	-0.342535000	2.495255000	1.702261000
H	-0.010816000	1.739332000	2.415100000
H	0.311396000	3.364206000	1.784592000

[Pt_3Cl_6{Te(CH_2)$_6$}$_4$] C_1 E=-5133.82851344 a.u.

Pt	3.320924000	-0.226714000	0.008568000
Pt	-0.869579000	-1.519234000	-0.060186000
Pt	-0.643947000	1.638979000	0.021760000
Te	1.482135000	-1.648664000	0.975738000
Te	-3.203688000	-1.426418000	-1.152774000
Te	-2.993066000	1.833247000	1.072322000
Te	1.715626000	1.436328000	-0.981840000
Cl	5.001649000	0.966308000	-1.047450000
Cl	4.838720000	-1.632045000	1.049603000
Cl	0.309214000	-1.633752000	-2.049264000
Cl	-2.025225000	-1.450154000	1.953295000
Cl	-1.753484000	1.786782000	-2.009437000
Cl	0.498442000	1.547345000	2.035481000
C	2.099841000	-3.643601000	0.579349000
H	2.242952000	-3.648475000	-0.501642000
H	3.081257000	-3.710661000	1.045638000
C	1.101583000	-4.687963000	1.044216000
H	1.628435000	-5.648845000	1.083546000
H	0.324763000	-4.792258000	0.281232000
C	0.413158000	-4.421381000	2.376660000
H	-0.257105000	-3.560233000	2.265762000
H	-0.255310000	-5.263797000	2.579692000
C	1.295604000	-4.220560000	3.603814000
H	1.915973000	-5.108829000	3.764168000
H	0.624960000	-4.161741000	4.467395000
C	2.202929000	-2.988022000	3.624024000
H	2.501171000	-2.803085000	4.662019000
H	3.139199000	-3.166671000	3.090944000
C	1.569889000	-1.709282000	3.111812000
H	2.158306000	-0.826126000	3.355465000
H	0.538816000	-1.558335000	3.436483000
C	-2.907273000	-1.879476000	-3.225953000
H	-3.594027000	-1.211088000	-3.746995000
H	-1.885507000	-1.550825000	-3.418564000
C	-3.119186000	-3.325585000	-3.623769000
H	-4.159014000	-3.621609000	-3.447720000
H	-2.990674000	-3.358186000	-4.711829000
C	-2.168350000	-4.338940000	-2.976814000
H	-1.835774000	-5.035661000	-3.750997000
H	-1.266148000	-3.825366000	-2.629410000
C	-2.765533000	-5.170959000	-1.846638000
H	-2.112554000	-6.033062000	-1.676234000
H	-3.725549000	-5.585908000	-2.179973000
C	-2.958611000	-4.481135000	-0.505741000
H	-3.320189000	-5.229008000	0.210314000
H	-1.994931000	-4.130833000	-0.120534000
C	-3.940258000	-3.325603000	-0.478946000
H	-4.222405000	-3.081463000	0.546679000
H	-4.852913000	-3.527993000	-1.044275000
C	-3.415723000	3.868644000	0.545255000
H	-3.658094000	3.755369000	-0.513219000
H	-4.329632000	4.148737000	1.074270000
C	-2.293717000	4.870448000	0.737301000
H	-2.497830000	5.716487000	0.070000000
H	-1.357583000	4.423314000	0.386879000
C	-2.111908000	5.416797000	2.144188000

H	-1.356214000	6.206940000	2.089461000
H	-3.039000000	5.911959000	2.461006000
C	-1.684689000	4.427745000	3.223297000
H	-1.332447000	5.013100000	4.077039000
H	-0.821039000	3.849050000	2.879604000
C	-2.776717000	3.473386000	3.718326000
H	-2.704172000	3.383582000	4.808392000
H	-3.766255000	3.904236000	3.530261000
C	-2.702045000	2.058894000	3.182715000
H	-3.467553000	1.417685000	3.621662000
H	-1.725921000	1.607031000	3.360685000
C	1.806736000	1.393621000	-3.109744000
H	2.504913000	0.582044000	-3.312535000
H	0.799368000	1.093232000	-3.400628000
C	2.232162000	2.704603000	-3.731434000
H	3.256471000	2.943354000	-3.430613000
H	2.279638000	2.518178000	-4.810177000
C	1.298821000	3.892879000	-3.465923000
H	0.305622000	3.527798000	-3.178051000
H	1.154273000	4.430257000	-4.406771000
C	1.797781000	4.906316000	-2.439177000
H	2.838205000	5.164686000	-2.670785000
H	1.223109000	5.829230000	-2.565508000
C	1.696419000	4.509632000	-0.974147000
H	0.650005000	4.311575000	-0.715254000
H	1.998502000	5.369894000	-0.365951000
C	2.555384000	3.340041000	-0.539043000
H	2.635036000	3.274727000	0.547588000
H	3.561347000	3.328251000	-0.958496000

$[Pt_4Cl_8\{Te(CH_2)_6\}_4$ D_2 E=-6173.45185839 a.u.

Pt	-4.082078000	0.000000000	0.000000000
Pt	0.000000000	1.537514000	0.000000000
Te	-2.376037000	-1.574905000	0.953694000
Te	-2.376037000	1.574905000	-0.953694000
Cl	-5.673083000	-1.342847000	1.007258000
Cl	-5.673083000	1.342847000	-1.007258000
Cl	-1.044504000	1.595352000	2.063720000
Cl	1.044504000	1.595352000	-2.063720000
C	-2.470431000	-1.573703000	3.081067000
H	-1.490885000	-1.197948000	3.378847000
H	-3.233489000	-0.824932000	3.291917000
C	-2.792164000	-2.925414000	3.677689000
H	-3.793588000	-3.239632000	3.369283000
H	-2.857911000	-2.758402000	4.758545000
C	-1.766944000	-4.032153000	3.400610000
H	-0.800364000	-3.585871000	3.138732000
H	-1.596200000	-4.577805000	4.331988000
C	-2.170020000	-5.059142000	2.345457000
H	-1.525597000	-5.935697000	2.463068000
H	-3.189339000	-5.404725000	2.555607000
C	-2.080245000	-4.628476000	0.889135000
H	-2.296454000	-5.500281000	0.261410000
H	-1.051492000	-4.334381000	0.649695000
C	-3.034708000	-3.534092000	0.456990000
H	-4.043896000	-3.618958000	0.860323000
H	-3.101099000	-3.454079000	-0.629550000
C	-2.470431000	1.573703000	-3.081067000
H	-1.490885000	1.197948000	-3.378847000
H	-3.233489000	0.824932000	-3.291917000
C	-2.792164000	2.925414000	-3.677689000
H	-3.793588000	3.239632000	-3.369283000
H	-2.857911000	2.758402000	-4.758545000
C	-1.766944000	4.032153000	-3.400610000
H	-1.596200000	4.577805000	-4.331988000
H	-0.800364000	3.585871000	-3.138732000
C	-2.170020000	5.059142000	-2.345457000
H	-1.525597000	5.935697000	-2.463068000
H	-3.189339000	5.404725000	-2.555607000
C	-2.080245000	4.628476000	-0.889135000
H	-2.296454000	5.500281000	-0.261410000
H	-1.051492000	4.334381000	-0.649695000
C	-3.034708000	3.534092000	-0.456990000
H	-4.043896000	3.618958000	-0.860323000
H	-3.101099000	3.454079000	0.629550000
Pt	4.082078000	0.000000000	0.000000000
Pt	0.000000000	-1.537514000	0.000000000
Te	2.376037000	1.574905000	0.953694000
Te	2.376037000	-1.574905000	-0.953694000
Cl	5.673083000	1.342847000	1.007258000
Cl	5.673083000	-1.342847000	-1.007258000
Cl	1.044504000	-1.595352000	2.063720000
Cl	-1.044504000	-1.595352000	-2.063720000
C	2.470431000	1.573703000	3.081067000
H	1.490885000	1.197948000	3.378847000
H	3.233489000	0.824932000	3.291917000
C	2.792164000	2.925414000	3.677689000
H	3.793588000	3.239632000	3.369283000
H	2.857911000	2.758402000	4.758545000
C	1.766944000	4.032153000	3.400610000
H	0.800364000	3.585871000	3.138732000
H	1.596200000	4.577805000	4.331988000
C	2.170020000	5.059142000	2.345457000
H	1.525597000	5.935697000	2.463068000
H	3.189339000	5.404725000	2.555607000
C	2.080245000	4.628476000	0.889135000
H	2.296454000	5.500281000	0.261410000
H	1.051492000	4.334381000	0.649695000
C	3.034708000	3.534092000	0.456990000
H	4.043896000	3.618958000	0.860323000
H	3.101099000	3.454079000	-0.629550000
C	2.470431000	-1.573703000	-3.081067000
H	1.490885000	-1.197948000	-3.378847000
H	3.233489000	-0.824932000	-3.291917000
C	2.792164000	-2.925414000	-3.677689000
H	3.793588000	-3.239632000	-3.369283000
H	2.857911000	-2.758402000	-4.758545000
C	1.766944000	-4.032153000	-3.400610000
H	1.596200000	-4.577805000	-4.331988000
H	0.800364000	-3.585871000	-3.138732000
C	2.170020000	-5.059142000	-2.345457000

H	1.525597000	-5.935697000	-2.463068000	H	1.051492000	-4.334381000	-0.649695000
H	3.189339000	-5.404725000	-2.555607000	C	3.034708000	-3.534092000	-0.456990000
C	2.080245000	-4.628476000	-0.889135000	H	4.043896000	-3.618958000	-0.860323000
H	2.296454000	-5.500281000	-0.261410000	H	3.101099000	-3.454079000	0.629550000

[PtCl$_2${Te(CH$_2$)$_6$}$_2$]$_2$ dimer, cis conformation C$_i$ E=-4094.19210670 a.u.

Pt	1.137936000	-0.240996000	-1.241450000	H	3.583609000	4.392421000	0.574166000
Te	2.689410000	-1.742826000	0.089688000	H	5.130206000	1.843897000	0.237009000
Te	1.758327000	1.875665000	0.027578000	H	5.522670000	3.080346000	-0.925286000
Cl	-0.173537000	1.250464000	-2.517272000	H	3.737048000	1.536795000	-1.754688000
Cl	0.562846000	-2.015503000	-2.664614000	H	3.220190000	3.222257000	-1.719337000
C	1.667062000	-3.620496000	0.195201000	H	0.849006000	-3.420738000	0.886199000
C	2.510801000	-4.788858000	0.660670000	H	1.262070000	-3.753923000	-0.808790000
C	4.920755000	-4.205315000	-0.038368000	H	2.837993000	-4.626104000	1.693577000
C	3.724127000	-5.153198000	-0.191472000	H	1.837038000	-5.653700000	0.695245000
C	5.124989000	-3.192227000	-1.159949000	H	4.035901000	-6.159356000	0.100641000
C	3.897977000	-2.395242000	-1.561332000	H	3.428281000	-5.227446000	-1.243998000
C	3.537367000	2.382366000	-1.098090000	H	5.840772000	-4.789314000	0.051657000
C	4.733927000	2.743975000	-0.242773000	H	4.826187000	-3.669066000	0.913988000
C	4.487979000	3.822690000	0.822062000	H	5.925767000	-2.508096000	-0.862557000
C	4.399006000	3.306390000	2.257091000	H	5.488567000	-3.712829000	-2.054258000
C	3.116913000	2.590545000	2.658237000	H	3.193490000	-2.955781000	-2.176380000
C	2.762108000	1.358552000	1.844332000	H	4.154761000	-1.492450000	-2.115707000
Pt	-1.137936000	0.240996000	1.241450000	H	-3.630342000	-0.740735000	-1.613709000
Te	-1.758327000	-1.875665000	-0.027578000	H	-2.021037000	-0.740792000	-2.353670000
Cl	0.173537000	-1.250464000	2.517272000	H	-3.215965000	-2.275244000	-3.702787000
Cl	-0.562846000	2.015503000	2.664614000	H	-2.272859000	-3.287989000	-2.645941000
C	-2.762108000	-1.358552000	-1.844332000	H	-5.253088000	-2.641098000	-2.435035000
C	-3.116913000	-2.590545000	-2.658237000	H	-4.532043000	-4.152256000	-2.938918000
C	-4.399006000	-3.306390000	-2.257091000	H	-5.307630000	-4.544953000	-0.782170000
C	-4.487979000	-3.822690000	-0.822062000	H	-3.583609000	-4.392421000	-0.574166000
C	-4.733927000	-2.743975000	0.242773000	H	-5.130206000	-1.843897000	-0.237009000
C	-3.537367000	-2.382366000	1.098090000	H	-5.522670000	-3.080346000	0.925286000
Te	-2.689410000	1.742826000	-0.089688000	H	-3.737048000	-1.536795000	1.754688000
C	-3.897977000	2.395242000	1.561332000	H	-3.220190000	-3.222257000	1.719337000
C	-5.124989000	3.192227000	1.159949000	H	-4.154761000	1.492450000	2.115707000
C	-4.920755000	4.205315000	0.038368000	H	-3.193490000	2.955781000	2.176380000
C	-3.724127000	5.153198000	0.191472000	H	-5.925767000	2.508096000	0.862557000
C	-2.510801000	4.788858000	-0.660670000	H	-5.488567000	3.712829000	2.054258000
C	-1.667062000	3.620496000	-0.195201000	H	-2.837993000	4.626104000	-1.693577000
H	2.021037000	0.740792000	2.353670000	H	-1.837038000	5.653700000	-0.695245000
H	3.630342000	0.740735000	1.613709000	H	-1.262070000	3.753923000	0.808790000
H	3.215965000	2.275244000	3.702787000	H	-0.849006000	3.420738000	-0.886199000
H	2.272859000	3.287989000	2.645941000	H	-3.428281000	5.227446000	1.243998000
H	5.253088000	2.641098000	2.435035000	H	-4.035901000	6.159356000	-0.100641000
H	4.532043000	4.152256000	2.938918000	H	-4.826187000	3.669066000	-0.913988000
H	5.307630000	4.544953000	0.782170000	H	-5.840772000	4.789314000	-0.051657000

Table S4. PBE0-D3/def2-TZVP optimized geometries of the $[Pt_nCl_{2n}\{Te(CH_2)_6\}_m]$ (n = 1-4; m = 2-4).

***cis*-$[PtCl_2\{Te(CH_2)_6\}_2]$**<br>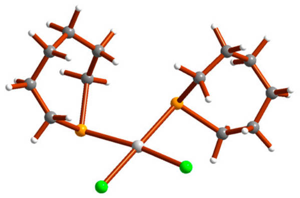<br>Pt-Te 2.528-2.531<br>Pt-Cl 2.326-2.339	***trans*-$[PtCl_2\{Te(CH_2)_6\}_2]$**<br>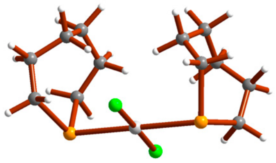<br>Pt-Te 2.583<br>Pt-Cl 2.315
**$[Pt_2Cl_4\{Te(CH_2)_6\}_3]$**<br>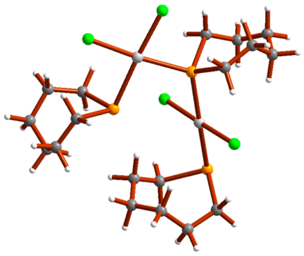<br>Pt-Te(*cis*) 2.501-2.543<br>Pt-Te(*trans*) 2.565-2.580<br>Pt-Cl(*cis*) 2.307-2.339<br>Pt-Cl(*trans*) 2.301-2.325<br>Te···Cl 3.636	**$[Pt_3Cl_6\{Te(CH_2)_6\}_4]$**<br>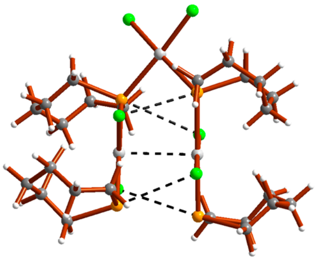<br>Pt-Te(*cis*) 2.515-2.518<br>Pt-Te(*trans*) 2.572-2.581<br>Pt-Cl(*cis*) 2.316<br>Pt-Cl(*trans*) 2.315-2.323<br>Pt···Pt 3.167<br>Te···Cl 3.508-3.628
**$[Pt_4Cl_8\{Te(CH_2)_6\}_4]$**<br>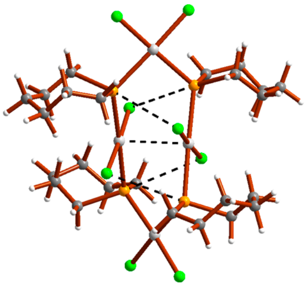<br>Pt-Te(*cis*) 2.510<br>Pt-Te(*trans*) 2.561<br>Pt-Cl(*cis*) 2.313<br>Pt-Cl(*trans*) 2.314<br>Pt···Pt 3.075<br>Te···Cl 3.613	

3.2 Secondary bonding interactions

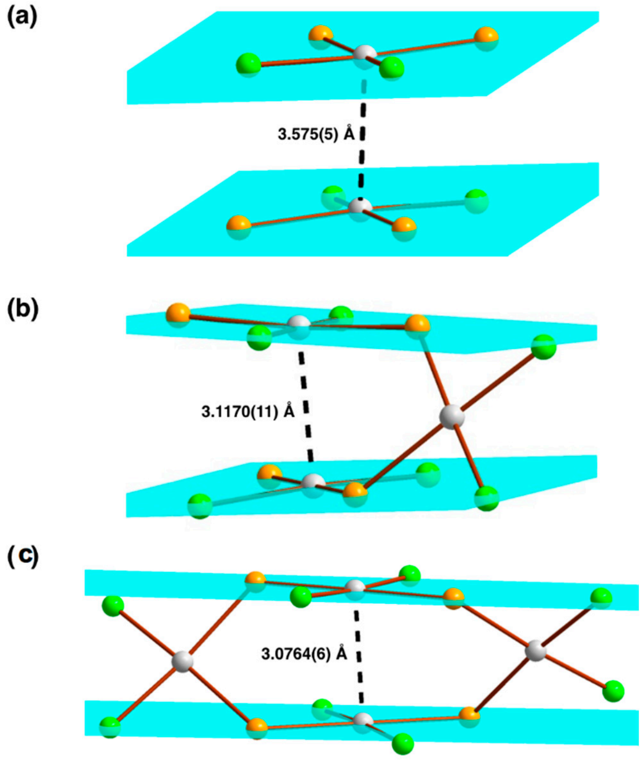

Figure S4. The Pt···Pt interactions lead to the square-planar coordination plane to become slightly concave in (a) $\mathbf{1}_{cis}$, (b) **2**, and (c) **3**.

3.3 Formation energetics

Table S5. Total energies of optimized species at PBE0-D3/def2-TZVP level of theory in vacuum (Hartree).

Species	*E*	*H*	G_{298}
cis-$[PtCl_2(NCPh)_2]$	-1688.06795	-1687.84233	-1687.91708
trans-$[PtCl_2(NCPh)_2]$	-1688.07709	-1687.85113	-1687.92435
PhCN	-324.22847	-324.12182	-324.15846
$Te(CH_2)_6$	-503.70425	-503.52586	-503.56845
cis-$[PtCl_2\{Te(CH_2)_6\}_2]$	-2047.06703	-2046.69856	-2046.77953
trans-$[PtCl_2\{Te(CH_2)_6\}_2]$	-2047.08132	-2046.71280	-2046.79224
$[Pt_2Cl_4\{Te(CH_2)_6\}_3]$	-3590.44077	-3589.88192	-3589.99880
$[Pt_3Cl_6\{Te(CH_2)_6\}_4\}]$	-5133.82851	-5133.07979	-5133.23107
$[Pt_4Cl_8\{Te(CH_2)_6\}_4\}]$	-6173.45186	-6172.69360	-6172.85587
$[PtCl_2(NCPh)\{Te(CH_2)_6\}]$	-1867.57576	-1867.27869	-1867.35713

Table S6. Total energies of optimized species at PBE0-D3/def2-TZVP level of theory in dichloromethane (Hartree).

	E	*H*	G_{298}
cis-$[PtCl_2(NCPh)_2]$	-1688.09774	-1687.87197	-1687.94624
trans-$[PtCl_2(NCPh)_2]$	-1688.10200	-1687.87599	-1687.94883
PhCN	-324.23565	-324.12893	-324.16554
$Te(CH_2)_6$	-503.70723	-503.52904	-503.57170
cis-$[PtCl_2\{Te(CH_2)_6\}_2]$	-2047.09060	-2046.72240	-2046.80539
trans-$[PtCl_2\{Te(CH_2)_6\}_2]$	-2047.09323	-2046.72499	-2046.80445
$[Pt_2Cl_4\{Te(CH_2)_6\}_3]$	-3590.46723	-3589.90901	-3590.02745
$[Pt_3Cl_6\{Te(CH_2)_6\}_4\}]$	-5133.85842	-5133.11156	-5133.26138
$[Pt_4Cl_8\{Te(CH_2)_6\}_4\}]$	-6173.49395	-6172.73679	-6172.90005
$[PtCl_2(NCPh)\{Te(CH_2)_6\}]$	-1867.59427	-1867.29725	-1867.37482

Table S7. Gibbs PBE0-D3/def2-TZVP formation energies of $\mathbf{1}_{cis}$, $\mathbf{1}_{trans}$, **2**, and **3** from *cis*-$[PtCl_2(NCPh)_2]$ and $Te(CH_2)_6$. In dichloromethane (kJ mol^{-1})-

Reaction	ΔE	ΔH	ΔG_{298}	ΔG/Pt	ΔG/Te
cis-$[PtCl_2(NCPh)_2]$ + 2 $Te(CH_2)_6$ ⇌ cis-$[PtCl_2\{Te(CH_2)_6\}_2]$ ($\mathbf{1}_{cis}$) + 2 PhCN	–130	–132	–123	-123	-61
cis-$[PtCl_2(NCPh)_2]$ + 2 $Te(CH_2)_6$ ⇌ trans-$[PtCl_2\{Te(CH_2)_6\}_2]$ ($\mathbf{1}_{trans}$) + 2 PhCN	–137	–139	–121	-121	-60
2 *cis*-$[PtCl_2(NCPh)_2]$ + 3 $Te(CH_2)_6$ ⇌ $[Pt_2Cl_4\{Te(CH_2)_6\}_3]$ + 4 PhCN	–243	–246	–215	-107	-71
3 *cis*-$[PtCl_2(NCPh)_2]$ + 4 $Te(CH_2)_6$ ⇌ $[Pt_3Cl_6\{Te(CH_2)_6\}_4]$ (**2**) + 6 PhCN	–394	–402	–339	-113	-85
4 *cis*-$[PtCl_2(NCPh)_2]$ + 4 $Te(CH_2)_6$ ⇌ $[Pt_4Cl_8\{Te(CH_2)_6\}_4]$ (**3**) + 8 PhCN	–418	–431	–401	-100	-100

4. References

[1] Rodewald, M.; Rautiainen, J. M.; Niksch, T.; Görls, H.; Oilunkaniemi, R.; Weigand, W.; Laitinen, R. S. Chalcogen-bonding interactions in telluroether heterocycles $[Te(CH_2)_m]_n$ (n = 1-4; m = 3-7). *Chem. Eur. J.* **2020**, *26*, 13806- 13818. doi.org/10.1002/chem.202002510
